# A global meta‐analysis of non‐lethal mammal browsing control in forests

**DOI:** 10.1002/brv.70205

**Published:** 2026-08-23

**Authors:** James B.H. Grimsdale, Daniel W.A. Noble, Geoffrey M. While, Dean Williams, Julianne O'Reilly‐Wapstra

**Affiliations:** ^1^ School of Natural Sciences University of Tasmania PO Box 805 Sandy Bay TAS 7006 Australia; ^2^ Division of Ecology and Evolution, Research School of Biology The Australian National University Canberra ACT 2601 Australia; ^3^ Centre for Sustainable Resource and Product Solutions University of Tasmania PO Box 805 Sandy Bay TAS 7006 Australia

**Keywords:** meta‐analysis, wildlife management, forests, forestry, conservation, landscape restoration, browsing, non‐lethal

## Abstract

Increasing resource demand, desire to sequester atmospheric carbon, and conservation goals are driving a need for forests to be managed more effectively in the face of numerous challenges. One such challenge is damage perpetrated by browsing mammals, which can cause substantial financial and ecological impacts. Current practices focus on lethal control measures for mitigating mammal browsing damage, however stakeholder pressure is calling for a transition to non‐lethal alternatives. Little is known about the relative efficacy of different non‐lethal control methods for mitigating browsing damage by mammals other than deer. Using a multilevel meta‐analytical framework across 1,908 effect sizes, we investigate the relative efficacy and longevity of different non‐lethal control methods implemented against a broad suite of browsing mammals across different spatial scales. We found that tree guards, repellents and exclusion fencing all significantly reduce mammal browsing damage and retain their effect for long periods. There was, however, substantial variation in efficacy within individual control strategies highlighting the importance to forest managers of selecting the correct method from within the broad strategies we identified. We then discuss other factors to consider when implementing non‐lethal mammal browsing control so that forest managers are well informed when implementing a control strategy.

## I. INTRODUCTION

Forest ecosystems cover over four billion hectares of the Earth's surface providing habitat for thousands of vertebrate species, food for hundreds of millions of people and timber for construction and generation of other wood products like paper (FAO and UNEP, 2020). Over one billion hectares are managed primarily for timber production with a further 749 million hectares under multi‐use management. An additional, estimated 85 million hectares are devoted to orchards, palm plantations and other agroforestry operations which are essential for human food production (FAO and UNEP, 2020). With an expected increase in demand for wood products (FAO and UNEP, 2020) and food (van Dijk *et al*., 2021), and a decline in available space (Chen *et al*., 2020), a major challenge moving forward is managing these areas in a way that maximises productivity while ensuring the sustainability of multiple use landscapes for economic, environmental, and social values into the future.

Commercial forests face significant challenges that impact growth and productivity (Keenan, 2015; Lehmann *et al*., 2020; Sturrock *et al*., 2011). One major challenge is browsing by mammal species including megafauna (Siipilehto & Heikkilä, 2005), marsupials (Miller *et al*., 2008) and rodents (Lyly *et al*., 2018). The scale of the problem is vast. In, 2015, across 14 European countries representing only 19.4% of total European forest area, mammal browsing damage was reported in over 945,000 hectares of forest (Feretti *et al*., 2020). Heavy browsing increases seedling mortality (Neilsen & Pataczek, 1991) and can lead to crop failure. Even under light browsing pressure, where seedlings may recover, growth and form are impacted which increases the likelihood the seedling will be browsed again (Welch *et al*., 1991). These impacts are also common in restoration, rehabilitation, and conservation landscapes. Restoration *via* reforestation provides tangible biodiversity conservation benefits (Kemppinen *et al*., 2020) and presents an avenue for carbon sequestration that could slow the rate of climate change (Basuki *et al*., 2022; Ménard *et al*., 2023) but can be severely hindered by browsing mammals (Forsyth *et al*., 2015). Hence, reducing the impact of browsing mammals is critical for both production and sustainability goals.

To mitigate browsing by mammalian herbivores, a suite of control methods have been developed (Miller *et al*., 2009; Warburton *et al*., 2022). Lethal measures are often seen as the most effective way of managing mammals but, despite their prevalence, they are not universally successful (Courchamp, Chapuis & Pascal, 2003; Thurfjell, Ciuti & Boyce, 2017). Moreover, lethal control of mammals can be restricted by a lack of social license. Answering the question of whether lethal control is acceptable requires consideration of multiple factors (Boulet *et al*., 2021; Farnworth, Watson & Adams, 2014; Fix *et al*., 2010), including the species being controlled (Sinclair *et al*., 2019) and the demographics of the people co‐existing with those species (Dawson *et al*., 2024). This creates a complex landscape of social perceptions that land managers must monitor to ensure they maintain a social licence for their mammal control operations.

Debate around lethal control measures has led to a push for alternative management strategies, which have been successful in certain contexts (Mo *et al*., 2023). Due to the diverse types of damage caused by browsing mammals including bark stripping (Nantongo *et al*., 2022), leaf and stem consumption and rubbing (Truax *et al*., 2021) a wide range of non‐lethal browsing mitigation strategies have been developed. These include fencing to prevent animals from accessing plants (e.g. Love, Bork & Spaner, 2009), chemical repellents to make plants unpalatable (e.g. Curtis & Boulanger, 2010) and acoustic deterrents to deter animals from planted areas (e.g. McGann *et al*., 2024). Additionally, plants can be manipulated to reduce the amount of browsing by altering their physical characteristics (e.g. Yagi, 2022), changing fertiliser regimes to alter nutritional value of plants (e.g. Brinks *et al*., 2011) or identifying genetic variants that are more resistant to browsing damage (e.g. O'Reilly‐Wapstra, McArthur and Potts, 2004). Given the diversity of non‐lethal control strategies, it is important to understand which methods are most effective at reducing browsing impacts. Both for encouraging a shift away from lethal control and for facilitating development of novel non‐lethal strategies (Eason *et al*., 2017).

Previous work has examined the impact of non‐lethal mammal control methods, but this has largely been limited to a single browsing taxon within a specific control context (Redick & Jacobs, 2020), or to a single control method applied to a single browsing taxon (Guerisoli & Pereira, 2020). There has been little effort to compare the relative effectiveness of different non‐lethal mammal control methods across multiple browsing mammal taxa and forestry systems. We fill this knowledge gap by investigating the relative efficacies of different non‐lethal mammal control methods across different browsing taxa and across spatial and temporal scales using a large meta‐analytical approach across multiple forestry systems. To achieve this, we extracted data on the impact of different non‐lethal mammal control strategies from 156 studies and used this data to answer four core questions: (*i*) Do different non‐lethal control methods reduce mammal browsing equally?; (*ii*) Is there variation in our ability to control browsing by different mammal taxa?; (*iii*) Do non‐lethal mammal control methods retain their efficacy over time?; and (*iv*) Does spatial scale impact the relative effectiveness of non‐lethal controls?

Based on our findings, we provide suggestions for land managers, combining our results with practical considerations, to enable identification of optimal non‐lethal mammal control techniques for implementation and identification of gaps in the non‐lethal control arsenal that need to be filled.

## II. MATERIALS AND METHODS

### (1) Literature synthesis

Our systematic literature search is reported following the preferred reporting items for systematic reviews and meta‐analyses (PRISMA) (Page *et al*., 2021). We searched the Web of Science (WoS) (‘All Databases’) and Scopus on 3 June 2024 for peer‐reviewed literature published in English from 1940 to present using the ‘Topic’ search term: (mammal OR marsupial OR ungulate OR deer OR macropod OR possum OR wallaby OR kangaroo) AND (audio OR sound OR laser OR fencing OR repellent OR deterrent OR non‐lethal) AND (brows*) AND (control OR damage).

All titles and abstracts were read as an initial screening. Our WoS search resulted in 397 papers. One hundred eighty four papers were excluded at the title and abstract screening stage, including four reviews. Scopus produced 118 results; 95 were duplicates from WoS, 12 were excluded at the title and abstract screening. Papers in the cited literature of the four reviews identified in the WoS search (Adams, 1975; Guerisoli & Pereira, 2020; Nolte & Wagner, 2000; Redick & Jacobs, 2020) were considered; 28 papers passed title and abstract screening. This created a shortlist of 252 papers that were read in full. After full text screening a further 68 papers were excluded leaving 184 papers for data extraction (see online Supporting Information – Appendix S1, Fig. S1 for a PRISMA flow diagram of the screening process). A further 28 papers were excluded during data extraction due to key data not being available (see sections II.2 and II.7 below).

Throughout, papers were included based on specific criteria: (*i*) *An experimental manipulation must have been applied* – a non‐lethal control measure must have been applied to protect against mammal browsing damage. An untreated control must have been established to enable comparison; (*ii*) *Non‐lethal control measures must have been related to trees* – the impacts of non‐lethal measures such as fencing and tree guards are easily related to the trees they protect. However, studies testing repellents had a range of experimental designs. Studies where the repellents were applied and the related trees measured (e.g. Andelt, Burnham & Manning, 1991) were included. Studies that tested repellence by applying compounds to a pellet diet (e.g. Arnould & Signoret, 1993) or to an unplanted area (e.g. Cox *et al*., 2012) and measuring aversion behaviours were excluded as repellents that may be effective at deterring access to a pellet diet or open area may not retain their efficacy if seedlings are present altering the patch quality; and (*iii*) *Measured variables needed to have a clear and direct link to browsing* – many studies quantified browsing using plant traits, such as height or percentage foliage removed, where changes can be readily attributed to changes in mammal browsing pressure. In contrast, variables such as species diversity are more difficult to interpret, as increases or decreases cannot be unambiguously classified as an improvement or worsening of browsing damage. To avoid this ambiguity, data based on species diversity or equivalent indirect measures were excluded.

### (2) Data extraction and transformation

From the 184 papers included in the full text screening we extracted either: (*i*) the mean, sample size and estimate of variation for control and treatment groups (Type 1) and/or (*ii*) the sample size and proportion that exhibited a given response in the control and treatment group (Type 2). For Type 1 data all estimates of variation were converted to standard deviations. Type 1 data with a bounded distribution (such as mean percentage survival measured across multiple plots) were logit transformed to satisfy normality assumptions per Harrison, Noble & Jennions (2022). Type 2 data presented only the raw proportions and sample sizes. Different effect sizes were calculated for Type 1 and Type 2 data and then converted to a common metric (See Section II.3 below).

Data were extracted from text, tables, figures or supplementary files. Data in figures were extracted using the R package *shinyDigitise* (Ivimey‐Cook *et al*., 2023). Where adjusted means and raw data were reported, the raw data was used; where raw data was unavailable, adjusted means were excluded. Where data was not available for a particular study (e.g. raw data or summary statistics), we contacted authors for clarification. In instances where sample sizes were not available but other data, such as the number of seedlings planted and survival, was reported sample sizes were calculated from available information. Where relevant information could not be supplied effects were excluded with some exceptions, see Section II.7.

### (3) Effect size calculation

Browsing outcomes were measured in several ways (e.g. seedling height, percentage foliage removal, seedling density) and so we derived a standardised effect size that allowed us to compare within and across studies. Effect sizes were calculated using R package *metafor*, (version 4.6–0; Viechtbauer, 2010). The standardised mean difference (SMD) (Hedges & Olkin, 2014) was calculated from type 1 data whereas the log odds ratio (Borenstein *et al*., 2021) was calculated for type 2 data and then converted using Equations 1 and 2 to SMD as follows (Borenstein, 2009):

(1) $$\mathrm{SMD}=\left(\mathrm{Log}\;\text{odds ratio}\times \sqrt{3}\right)\times \pi$$

(2) $$\mathrm{SMD}\;\text{variance}=\left(\mathrm{Log}\;\text{odds ratio variance}\times 3\right)÷\pi^{2}$$

SMD was selected over the commonly used log response ratio because log odds ratios can be converted to SMD but log response ratios cannot. Due to the breadth of variables measured across studies there were instances where a reduction in browsing was denoted by both positive and negative effect sizes. To facilitate easy comparison, the sign of the effect sizes was switched so that a reduction in browsing was always denoted by a positive effect size. Input data for effect sizes of a magnitude greater than 10 were checked in the original source material.

### (4) Moderator variables

We extracted data on key moderator variables, which (*i*) we predicted would explain variation in the efficacy of non‐lethal browsing control; (*ii*) were of interest for land management; or (*iii*) were important covariates.

#### (a) Fixed effects

There are three types of fixed effects. The first is the *type of control method* – the broad category of control method that was implemented. We expected there to be substantial variation between different control strategies. Methods were grouped into seven broad categories: exclusion fence, other fencing, repellents, tree guards, neighbour effects, natural debris and stock manipulation.

Secondly, *browsing fauna* was a fixed effect representing the taxon of browsing mammal that was being controlled. We predicted that mitigation of browsing by different groups of mammals would vary based on different browsing behaviours, forage preferences or morphology. Browsing fauna were recorded as deer, other ungulates, other, mixed or not specified. Other includes all non‐ungulate browsers such as lagomorphs and marsupials. Mixed denotes studies that specified multiple browsing species that would fall into different groups. We opted for these broad taxonomic groupings to ensure sample sizes in each group were sufficient to draw robust conclusions. Effects investigating deer were ubiquitous (*k* = 1,059), hence its inclusion as a standalone group.

The third fixed effect was *Control method duration* – the length of the interval between establishing the control method and the collection of data. We hypothesised that there would be variation in how well different control methods retained their efficacy over time and the longevity of browsing mitigation is of high importance for land managers. Many studies (*N* = 84) used repeated measures of the same experimental units which increases the non‐independence of effects. In these instances, up to three effects for each pairwise comparison were included with time points selected at first measurement, last measurement and an intermediate measurement as close to the median time as was available.

#### (b) Covariates

*Plot size* is a covariate for the size of the plots that were being measured. Plot size was extracted where possible as it has been shown to influence effect size and failure to account for this can bias the results of ecological meta‐analyses (Spake *et al*., 2021). Where plot size was not described (*k* = 352) a value was imputed (See Section II.7).

#### (c) Random effects

Additionally, three random effects were included. The first, *Plant genus*, records the genus of the plants that were being protected from browsing by a non‐lethal control. We predicted that effect sizes would differ based on plant genus due to variation in browsing defences. Where plant genus was not specified or multiple genera were specified the genus was classified as various. Genus was chosen over species as the taxonomic level of replication to reduce the number of levels within this factor and avoid issues associated with small sample sizes.

Second, *Cohort ID* was a shared ID for all effects generated by measurement of the same experimental unit. Eighty four studies used repeated measurements of the same experimental units (i.e. the same plants or plots measured at multiple points in time), which creates non‐independence. Effects based on measurements of the same experimental units used a shared cohort ID to account for this.

Third, a *Study ID* was assigned as a shared ID for effects originating from the same study. Even if the same experimental units were not being measured there is non‐independence created between effects generated from data from the same study. We used a shared study ID to account for this.

### (5) Statistical analysis

Experiments were carried out at three different scales – the plant scale, the site scale and the landscape scale (Fig. 1). To test how different scales affect the efficacy of non‐lethal control strategies we analysed these biological scales separately. At each experimental scale our analysis followed the same protocol.

**Fig. 1:**
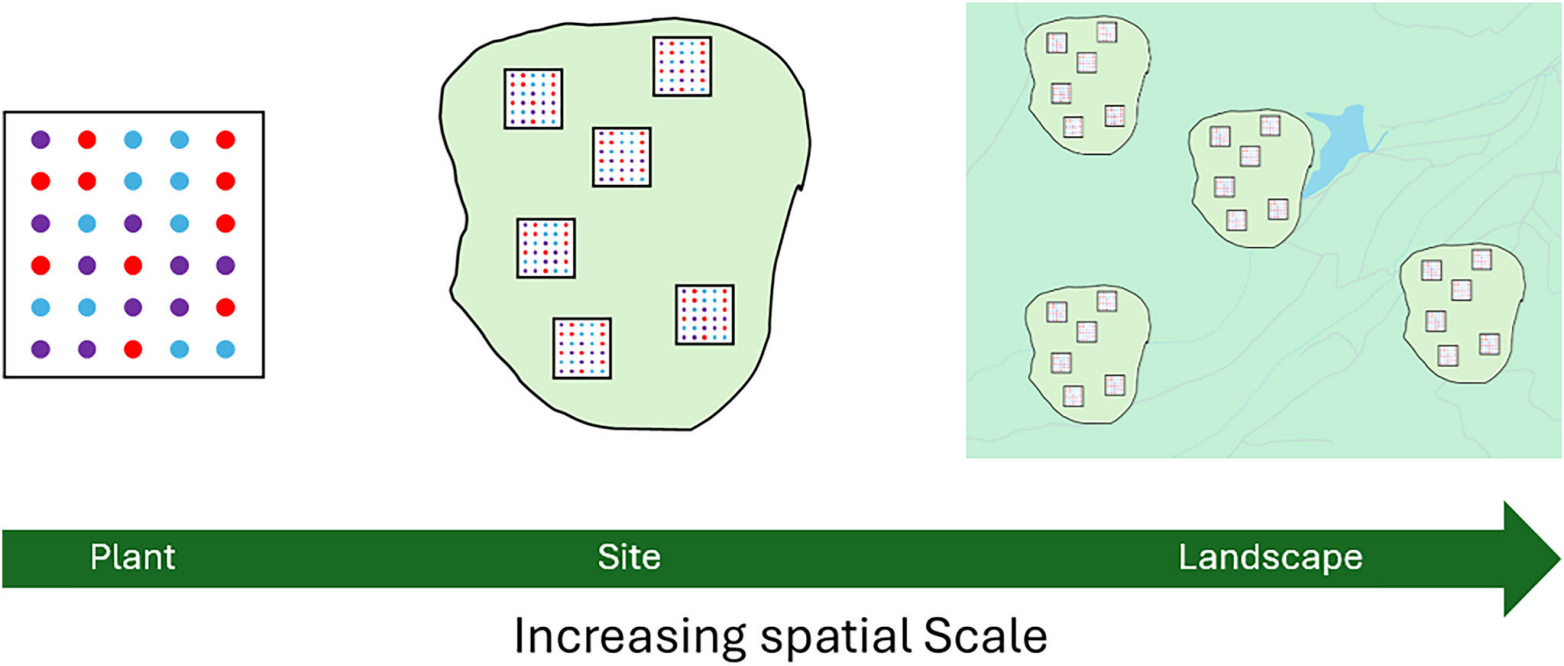
Experiments were categorised into three spatial scales: plant – a single plot with multiple trees, each randomly assigned to a treatment or control; site – multiple ‘plant scale’ plots spread across a single location, or multiple plots spread across a location where each plot is randomly allocated to a treatment or control; landscape – multiple ‘site scale’ locations spread across the broader landscape. Effect sizes at each scale were modelled separately.

First, to account for shared controls, we generated a variance–covariance matrix assuming effects sharing common control or treatment data were correlated by *r* = 0.5 (Appendix S1, Fig. S2). This allowed us to calculate a shared sampling covariance which was included in the sampling (co)variance matrix that was used in our models. Second, we included study level, cohort ID, and plant genera random effects. These accounted for multiple effect sizes coming from the same study, repeated measurement of the same experimental units and variation in browsing defences between plant genera. Finally, an observation‐level random effect was included to estimate residual variance.

To estimate overall mean effects we fit an intercept only multi‐level meta‐analytic (MLMA) model with the random effects detailed above. This model provided an overall effect of browsing control for each scale and allowed us to estimate sources of heterogeneity. We report four metrics of heterogeneity: prediction intervals (IntHout *et al*., 2016), coefficient of variation (CV) and its transformation (M), and *I* ^2^ estimates (Yefeng, Y., Noble, D.W.A., Spake, R., Senior, A.M., Lagisz, M. & Nakagawa, S., in preparation). Total *I* ^2^ indicates the overall proportion of effect heterogeneity removing sampling variance (Nakagawa & Santos, 2012). We also estimated the proportion of variance explained by the various random effects denoted as *I* ^2^_study_ (proportion of variance in effect explained by study differences) and *I* ^2^_genus_ (proportion of variance in effect explained by different plant genera). CV represents the magnitude of heterogeneity in relation to the mean effect – we used total CV; a measure of total variance as a proportion of the mean effect size. M is a measure of the magnitude of heterogeneity in context of the mean effect size using a transformation of the coefficient of variation – we report total M, which can be expressed as M_total_ = CV_total_/(1 + CV_total_) (Table 1 in Yefeng, Y., Noble, D.W.A., Spake, R., Senior, A.M., Lagisz, M. & Nakagawa, S., in preparation).

We then fit multi‐level meta‐regression (MLMR) models. These models included the same random effects outlined above. Three MLMR models were fitted: (*i*) a model that included control method as a fixed effect to assess whether there is a difference in efficacy between the different control methods; (*ii*) a model including browsing taxa type as a fixed effect to identify whether different browsers are more difficult to control; and (*iii*) a model accounting for control method, time and their interaction as fixed effects to assess how different control methods' effects vary over time. Model 3 used exclusion fencing as a baseline to contrast the other non‐lethal strategies against as it is known that exclusion fencing retains efficacy well over long time periods (Forsyth, 2023). All three MLMR models also included plot size as a fixed covariate. Control methods with a sample size smaller than 15 effects were removed from time series models. Any groupings that had a sample size smaller than 10 effects where all effects (*k*) originated from the same study (*N*) were removed from analysis (for example, guardian animal at a site scale; *k* = 3, *N* = 1).

### (6) Publication bias

We used two approaches to deal with, test for and correct for publication bias. First, we contacted the authors of all papers marked for data extraction, where an email could be sourced (*N* = 159/184), asking if there was any data collected during a study that was not included in the final manuscript. Fifty authors responded to our query and 15 provided us access to their raw data. Second, we evaluated the significance of both publication bias and time‐lag bias using the meta‐regression approach proposed by Nakagawa *et al*. (2022*a*). All models were refitted including fixed effects of the square root of the inverse of the effective sample size (see equations 26 and 27 in Nakagawa *et al*., 2022*a*) and centred publication year. Significant effects indicated support for publication bias. If publication bias was present, we provide bias corrected estimates (Nakagawa *et al*., 2022*a*).

### (7) Missing data and sensitivity analyses

Missing data is a common problem in meta‐analyses. In our case, we were missing data for two critical variables, the standard deviation for deriving some of our SMD values and plot size. Overall, we had missing data for a total of 887 effects (44 studies). While our main analysis used complete case data, excluding relevant data can reduce power. As such, we used multiple imputation to estimate a value of the standard deviation and plot size when this information was missing (Kambach *et al*., 2020; see Appendix S1 for further detail on the imputation process). We used the imputed data sets (*m* = 20) to conduct a sensitivity analysis with a greater number of effect sizes to test the robustness of our complete case analysis. To ensure that our analysis remained robust to non‐independence issues we also refit models using robust variance estimation (Nakagawa *et al*., 2022*b*). Instances where the three analyses diverge are highlighted in our results, otherwise the summary statistics of all analyses are included in the supplementary information (Appendix S1, Tables S1–S24).

## III. RESULTS

### (1) Data summary

In total, we extracted 1,908 effects from 112 papers across 21 different countries (Fig. 2) (*k* = 2,795 effects from 156 papers across 23 countries when considering imputed data). Most effects were presented at the site scale (*k* = 1,166), followed by the landscape and plant scales (*k* = 603 and *k* = 139 effect sizes, respectively). Exclusion fencing was the most common control measure (*k* = 674) used, with the major browsing mammal category being deer (*k* = 1,059).

**Fig. 2:**
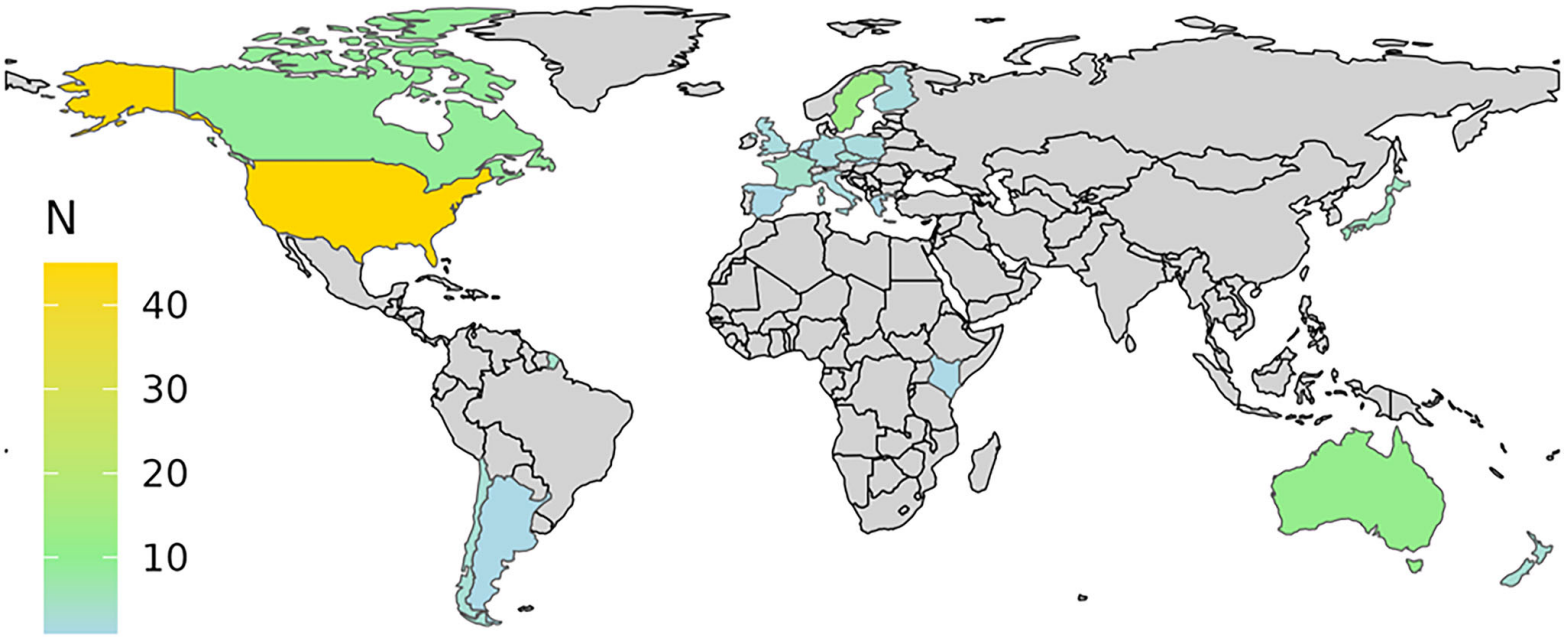
Experimental distribution map. Experiments were conducted in 21 countries globally with all continents except Antarctica represented. One study (Barrere *et al*., 2021) conducted experiments in two countries: France and Sweden.

### (2) Effects of non‐lethal browsing control at the plant scale

Overall, non‐lethal control measures suppressed browsing significantly at a plant scale [MLMA: overall meta‐analytic mean = 1.02, 95% confidence intervals (0.47, 1.58), *P* = 0.003, *k* = 139, *N* = 12; Table S1], but total heterogeneity was high (*I* ^2^_total_ = 90.2%, 95% prediction intervals = −1.47–3.52; CV = 1.03; M = 0.64; Table S25). Overall, 37.6% of variation was explained by study with a further 34.4% by cohort ID (Table S25).

When we explored the above effects in more detail using an MLMR, there was clear variation in the effectiveness of the two non‐lethal control methods used at the plant scale: repellents and exclusion fencing. Repellents significantly reduced mammal browsing, whereas exclusion fencing showed no detectable effect [MLMR: repellents: mean = 0.83 (0.14, 1.52), *P* = 0.03; exclusion fencing: mean = 0.28 (−3.71, 4.26), *P* = 0.87, *R* ^2^ = 82% (9%)] conditional (marginal) – *R* ^2^ values are quoted once for each MLMR model at each experimental scale; (Fig. 3A, Table S3). Effectiveness also varied among the three browsing taxa represented in the data at a plant scale. Non‐lethal control methods reduced browsing by deer, other ungulates, and other mammals (Fig. 3B), but this reduction was only statistically significant for other ungulates [mean = 1.65 (0.35, 2.96), *P* = 0.01, *R* ^2^ = 83% (12%); Table S5]. Only repellents had a sufficient sample size to analyse longevity; they retained their ability to mitigate browsing damage well but there was a slight, non‐significant reduction in efficacy over time [−0.47 (−1.63, 0.69), *P* = 0.42, *R* ^2^ = 85% (8%); Table S7] (Fig. 4).

**Fig. 3:**
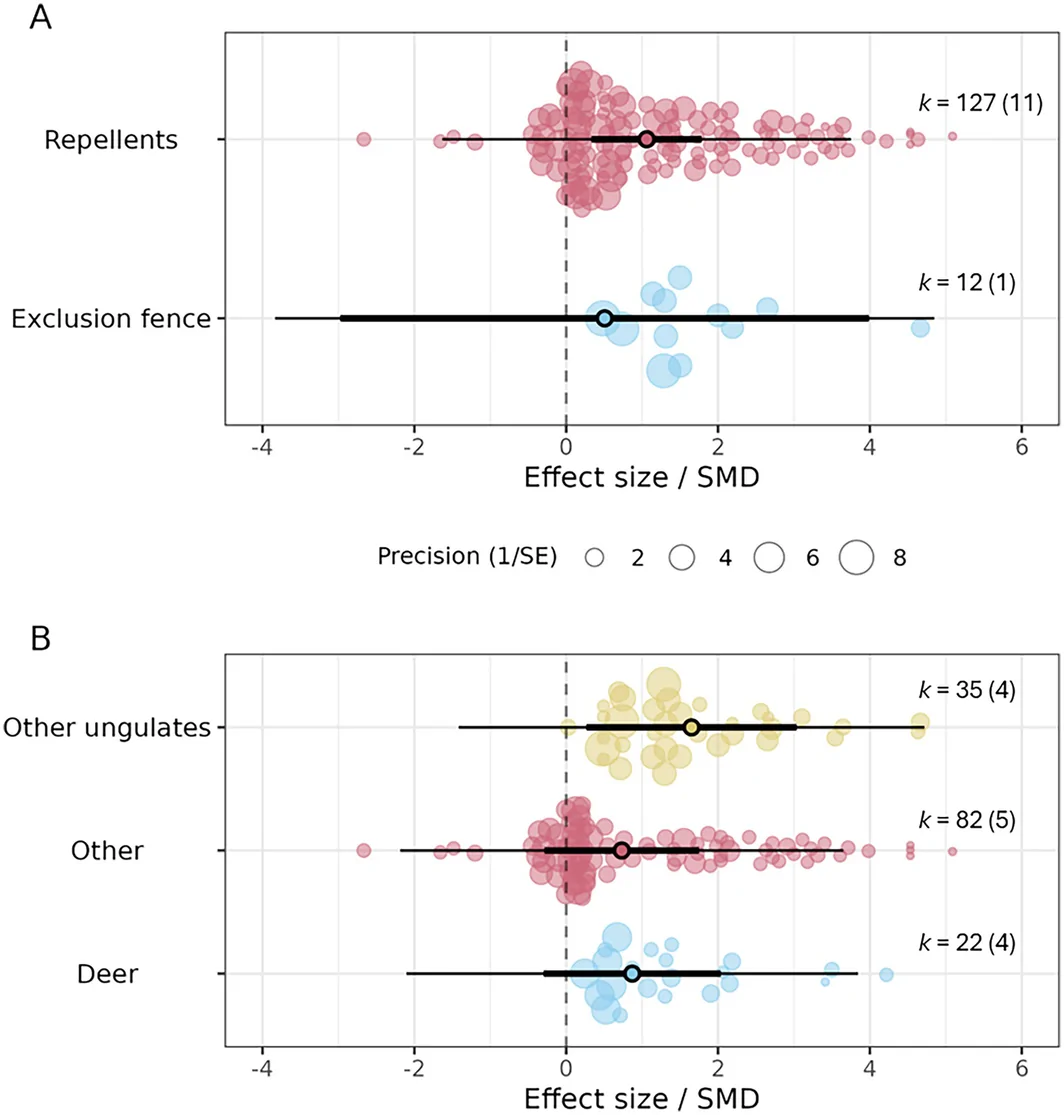
Orchard plots showing total effect sizes (k) across *n* studies, in brackets, for non‐lethal mammal browsing control at a ‘plant scale’. Bubbles represent individual effect sizes, increasing size denotes increasing precision; positive effect size represents a reduction in browsing damage. Points show marginalised mean effect size for different control methods (A) and different browsing taxa (B). Thick lines show 95% confidence intervals and thin lines show 95% prediction intervals. SMD = standardised mean difference.

**Fig. 4:**
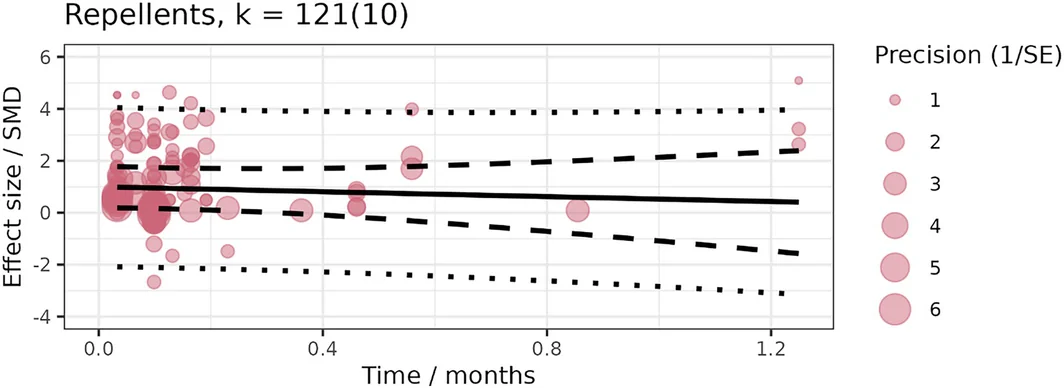
Bubble plot showing the retention of efficacy over time for Repellents at a ‘plant scale’. Number of effect sizes (k) and number of papers (in brackets) are shown in the plot title. The size of each bubble denotes the effect size precision. The solid line denotes the marginalised mean effect size. Dashed lines show the 95% confidence intervals and dotted lines show the 95% prediction intervals. SMD = standardised mean difference.

At a plant scale, there was no evidence for small sample size or time lag publication bias in our analysis (Tables S2, S4, S6 and S8).

### (3) Effects of non‐lethal browsing control at the site scale

Mammal browsing at a site scale was significantly reduced by non‐lethal control measures [MLMA: overall meta‐analytic mean = 0.64, (0.33, 0.95), *P* < 0.001, *k* = 1,166, *N* = 45; Table S9]. Total heterogeneity was high (*I* ^2^_total_ = 96.8%, 95% prediction intervals = −2.01 – 3.29; CV = 2.01; M = 0.80; Table S26). Cohort ID accounted for 38.7% of variation (Table S26).

Almost all non‐lethal browsing control strategies reduced mammal browsing at a site scale: stock manipulation and neighbour effects to a non‐significant extent and tree guards, repellents, natural debris and exclusion fences significantly (Fig. 5A, Fig. S3A), with natural debris and tree guards the two most effective strategies [MLMR: mean = 1.20 (0.36, 2.05), *P* = 0.005; mean = 1.20 (0.77, 1.63), *P* < 0.001 respectively; Table S11]. The only exception was neighbour effects, which increased browsing damage albeit not significantly [mean = −0.23 (−1.00, 0.53), *P* = 0.55, *R* ^2^ = 75% (7%); Table S11]. Non‐lethal control methods significantly reduced browsing in three of the five mammal groups: other ungulates [mean = 0.61 (0.03, 1.19), *P* = 0.04], deer [mean = 0.63 (0.20, 1.06), *P* = 0.004], and other mammals [mean = 0.88 (0.10, 1.65), *P* = 0.03; Fig. 5B, Fig. S3B, Table S13]. The largest reduction was observed for other mammals, although estimates for deer were more precise, as reflected by their narrower confidence intervals. Browsing by mixed mammal assemblages was reduced but not significantly [mean = 0.63 (−0.25, 1.51), *P* = 0.16].

**Fig. 5:**
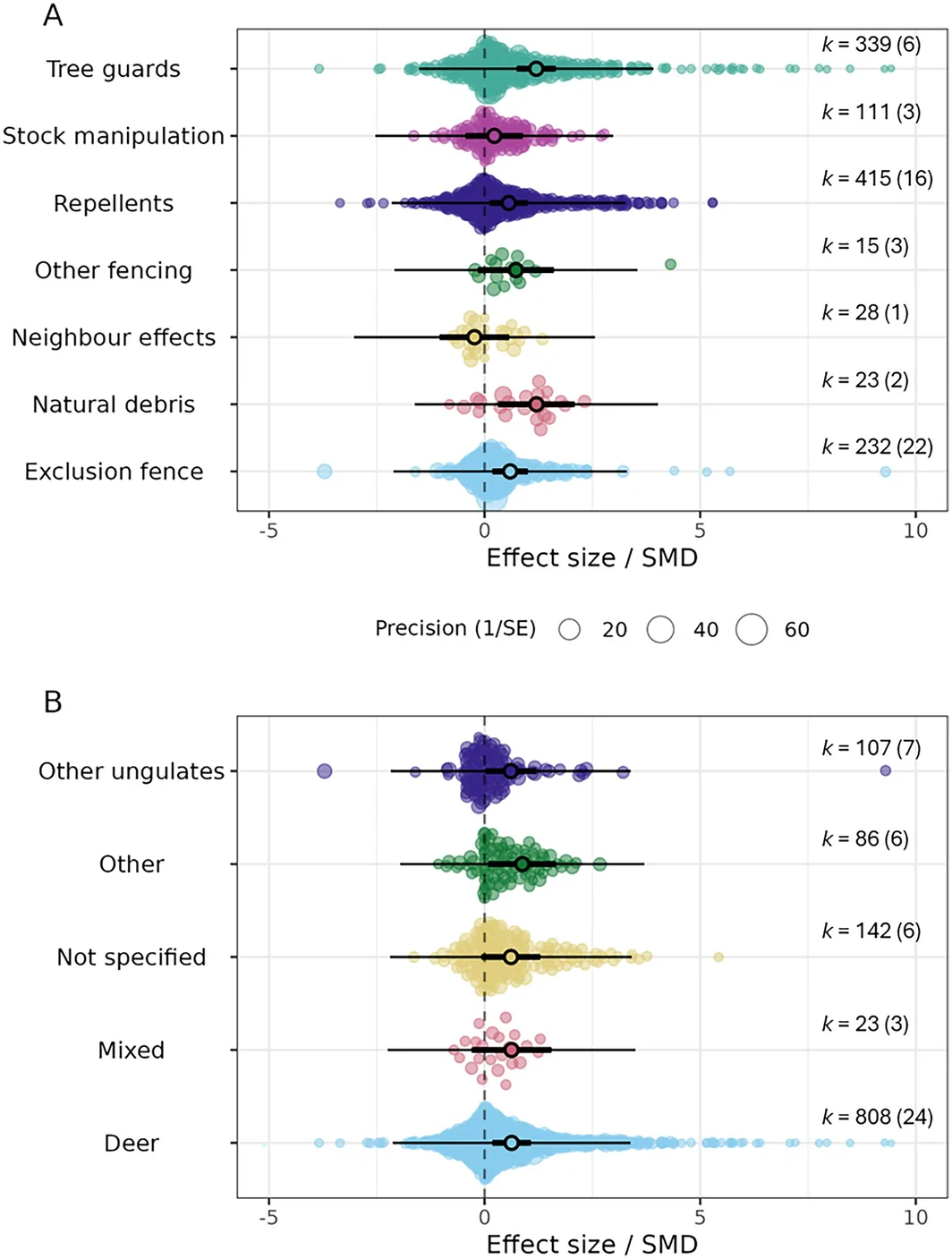
Orchard plots showing total effect sizes (*k*) across n studies, in brackets, for non‐lethal mammal browsing control at a ‘site scale’. Bubbles represent individual effect sizes, increasing size denotes increasing precision; positive effect size represents a reduction in browsing damage. Points show marginalised mean effect size for different control methods (A) and different browsing taxa (B). Thick lines show 95% confidence intervals and thin lines show 95% prediction intervals. Note the X axes have been limited for ease of viewing confidence and prediction intervals; an unedited figure is available in Fig. S2. SMD = standardised mean difference.

There was variation amongst non‐lethal control measures in their longevity. Tree guards retain their efficacy better than exclusion fencing but not to a significant extent [0.005 (−0.002, 0.01), *P* = 0.16] (Fig. 6; Table S15). All other methods showed reductions in efficacy relative to exclusion fencing but only repellents had their effect significantly reduced over time [−0.007 (−0.01, −0.00005), *P* = 0.048; Fig. S4A, Table S15]. Other fencing (including electric fences or plain or barbed wires without mesh aprons) showed the greatest loss of efficacy over time [−0.08 (−0.20, 0.03), *P* = 0.16]. Stock manipulation showed a small reduction in efficacy over time in our complete‐case analysis [− 0.002 (−0.01, 0.01), *P* = 0.70, *R* ^2^ = 80% (8%); Fig. S4B] but in our imputed‐case analysis this finding was reversed (0.01 se = 0.007, *P* = 0.06, see Table S15).

**Fig. 6:**
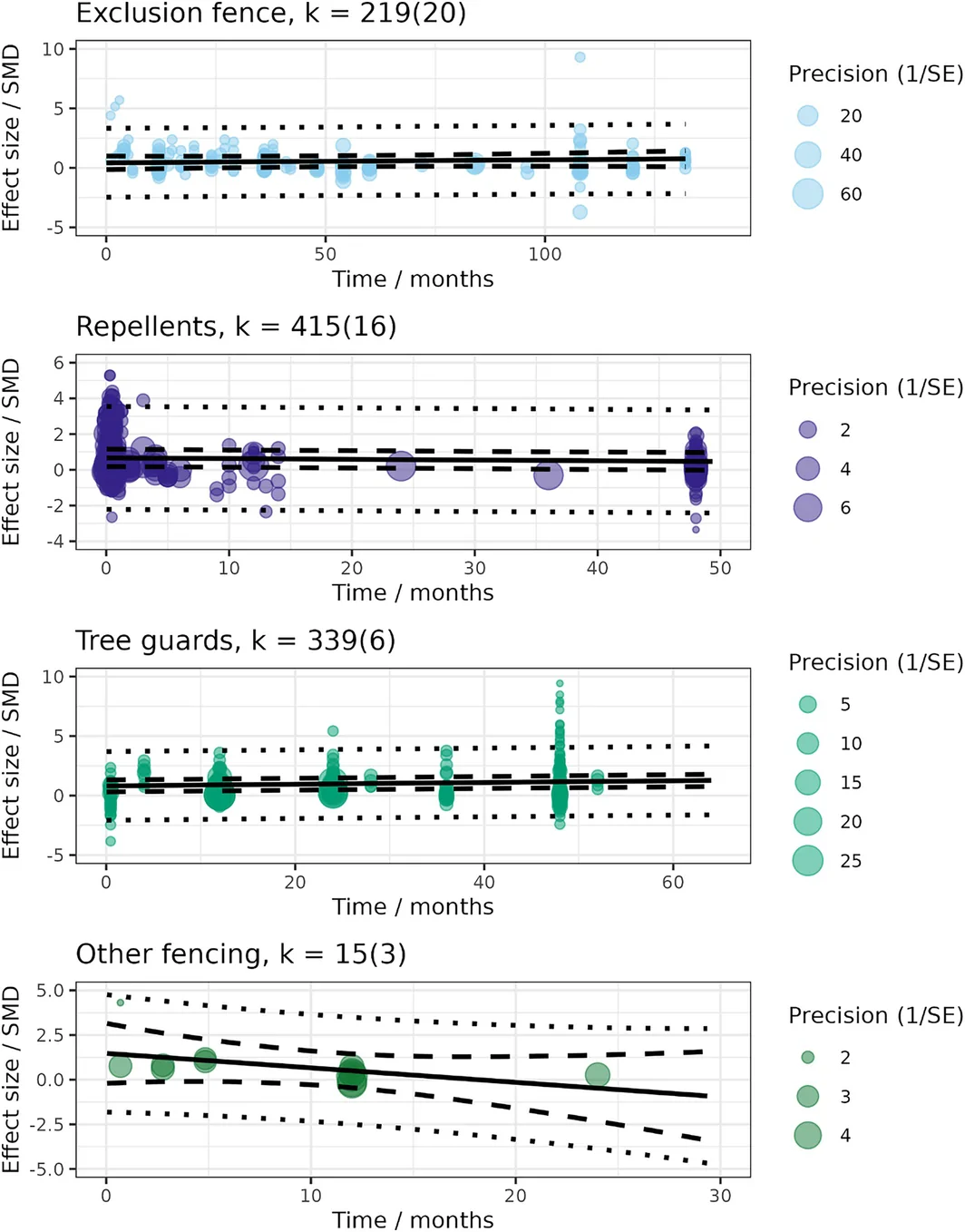
Bubble plots showing the retention of efficacy over time for selected non‐lethal mammal browsing control methods at a ‘site scale’. Methods, number of effect sizes (k) and number of papers (in brackets) are shown in subplot titles. The size of each bubble denotes the effect size precision. Solid lines denote the marginalised mean effect size. Dashed lines show the 95% confidence intervals and dotted lines show the 95% prediction intervals. Note the different subplot legends and different scales of the X and Y axes; Y axes have been limited to better show confidence and prediction intervals. Unedited figures showing all non‐lethal mammal browsing control methods implemented at a site scale are available in Figs. S3A and S3B. SMD = standardised mean difference.

There was no evidence for small sample size or time lag publication bias in any of our models at a site scale (Tables S10, S12, S14 and S16).

### (4) Effects of non‐lethal browsing control at the landscape scale

Non‐lethal control reduced mammal browsing significantly at a landscape scale [MLMA: overall meta‐analytic mean = 0.51 (0.35, 0.66) *P* < 0.001, *k* = 603, *N* = 57; Table S17]. Total heterogeneity, again, was high (*I* ^2^_total_ = 96.8%, 95% prediction intervals = −1.34 – 2.36; CV = 1.79; M = 0.73; Table S27). Cohort ID was responsible for 72.6% of variation with study explaining a further 18% (Table S27).

There was variation between the different non‐lethal control methods in the extent to which they altered browsing damage. Stock manipulation significantly increased browsing damage [mean = −1.59 (−2.39, −0.79), *P* < 0.001; Table S19] but this result is heavily influenced by an outlier. The other non‐lethal control methods all reduced mammal browsing on average, however only tree guards [mean = 0.76 (0.29, 1.24), *P* = 0.002], repellents (mean = 0.54 [0.04, 1.03], *P* = 0.03) and exclusion fencing (mean = 0.50 [0.29, 0.72], *P* < 0.001, *r* ^2^ = 95% [7%]) reduced browsing to a significant extent (Fig. 7A, Table S19). Browsing tended to be reduced across all mammal groups following non‐lethal control, however this reduction was only significant for deer (mean = 0.61 [0.34, 0.88], *P* < 0.001), other ungulates (mean = 0.32 [0.07, 0.57], *P* = 0.01) and unspecified browsing mammals (mean = 0.79 [0.36, 1.22], *P* < 0.001, *R* ^2^ = 95% [4%]) (Fig. 7B; Table S21).

**Fig. 7:**
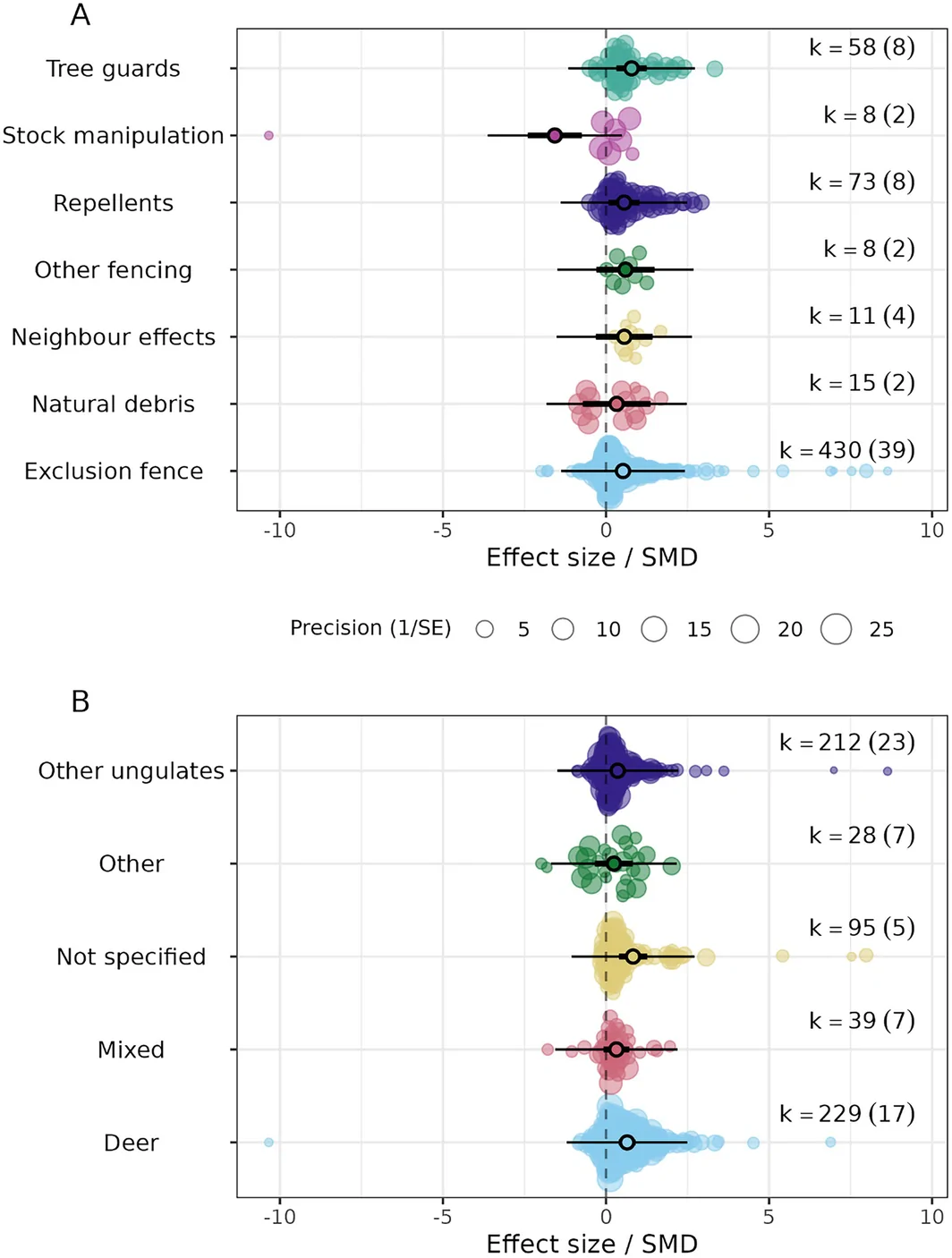
Orchard plots showing total effect sizes (k) across n studies, in brackets, for non‐lethal mammal browsing control at a ‘landscape scale’. Bubbles represent individual effect sizes, increasing size denotes increasing precision; positive effect size represents a reduction in browsing damage. Points show marginalised mean effect size for different control methods (A) and different browsing taxa (B). Thick lines show 95% confidence intervals and thin lines show 95% prediction intervals. SMD = standardised mean difference.

Longevity of browsing mitigation was analysed for four control methods. Relative to exclusion fencing only natural debris saw a significant loss of efficacy over time [−0.06 (−0.10, −0.03), *P* < 0.001], with repellents showing a non‐significant decrease in efficacy [−0.005 (−0.03, 0.01), *P* = 0.60] (Table S23). Tree guards retain their efficacy better than exclusion fencing but not to a significant extent [0.0004, (−0.006, 0.007), *P* = 0.91, *r* ^2^ = 96% (4%)] (Fig. 8; Table S23). Our imputed‐case analysis had sufficient sample size to model the longevity of browsing mitigation for neighbour effects; they lose efficacy over time but not at a significant rate relative to exclusion fencing (−0.01 se = 0.004, *P* = 0.61, supplementary Table S23).

**Fig. 8:**
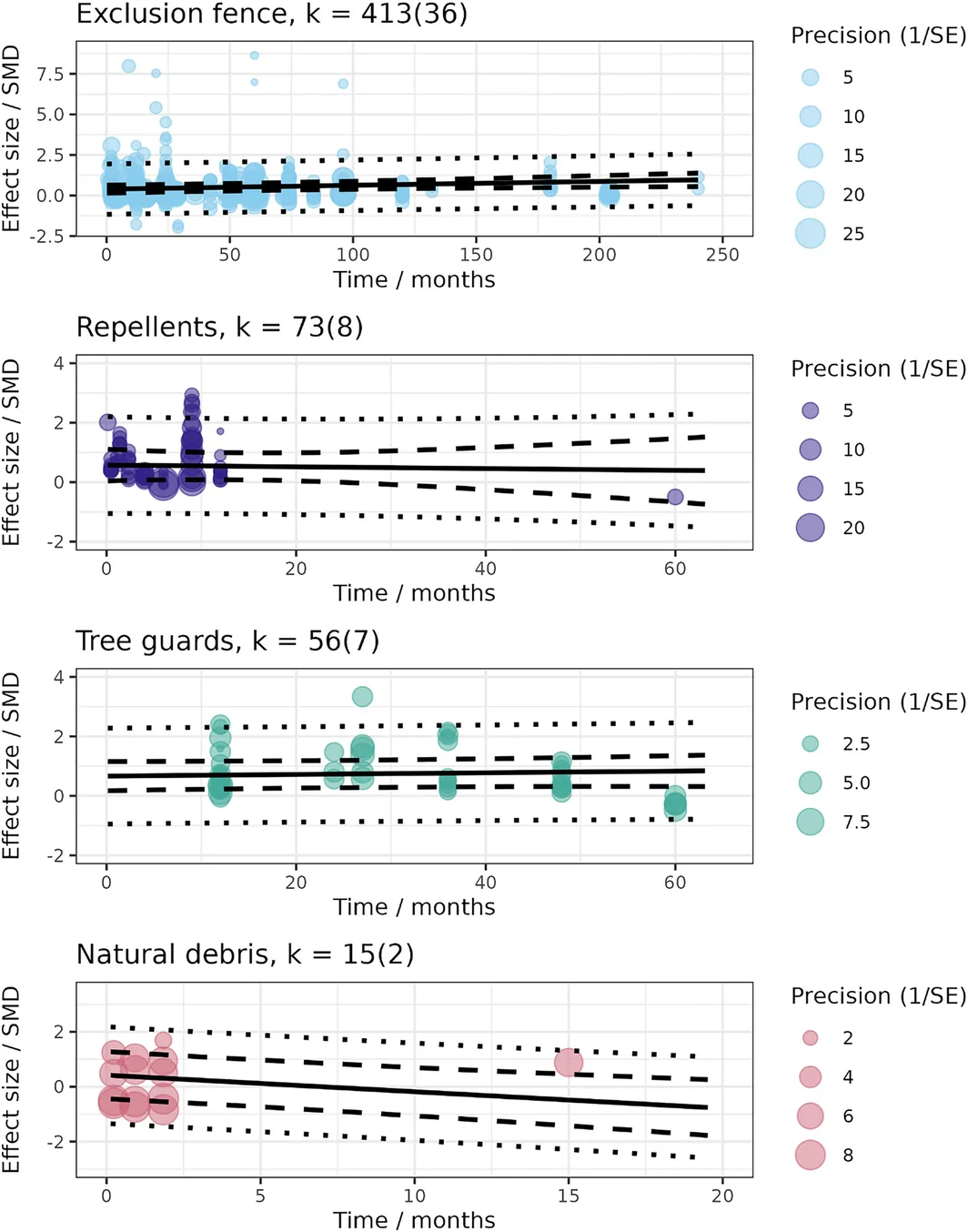
Bubble plots showing the retention of efficacy over time for different non‐lethal mammal browsing control methods at a ‘landscape scale’. Methods, number of effect sizes (k) and number of papers (in brackets) are shown in subplot titles. The size of each bubble denotes the effect size precision (see subplot legends). Solid lines denote the marginalised mean effect size. Dashed lines show the 95% confidence intervals and dotted lines show the 95% prediction intervals. Note the different subplot legends and different scales of the *X* and *Y* axes. SMD = standardised mean difference.

Our meta‐regression approach identified a significant effect of small sample size publication bias in our MLMR model interrogating the effect of browsing taxa on browsing mitigation [mean = 0.66 (0.06, 1.26), *P* = 0.03] (Table S22). Adjusted mean effect size estimates, 95% confidence intervals, standard errors and *P* values can be found in Table 1.

**Table 1:** Landscape scale multi‐level meta‐regression model results interrogating the impact of browsing taxa on efficacy of non‐lethal mammal browsing control, while accounting for time lag and small sample size publication bias. Effect sizes, confidence intervals and standard errors are adjusted for the significant (*P* < 0.05) impact of small sample size publication bias – represented here by ‘Effective sample size’. Positive effect sizes represent reduced browsing damage.

Term	Mean Effect size [95% confidence intervals]	Standard error	*P* value
Deer	0.459 [0.141, 0.777]	0.158	0.006
Mixed	0.095 [−0.335, 0.524]	0.219	0.665
Not specified	0.638 [0.158, 1.119]	0.239	0.010
Other	−0.052 [−0.698, 0.589]	0.319	0.871
Other ungulates	0.121 [−0.196, 0.438	0.161	0.455
Plot size	0.0002 [−0.0003, 0.0007]	0.0003	0.434
Publication year	0.001 [−0.014, 0.017]	0.008	0.879
Effective sample size	0.660 [0.063, 1.257]	0.304	0.030

## IV. DISCUSSION

We synthesised globally distributed literature – including that from some of the largest forest product exporting countries – to investigate how non‐lethal mammal control strategies influence browsing damage, the consistency of these effects across mammal taxa and their retention across spatial and temporal scales. Overall, multiple non‐lethal control strategies significantly reduced mammal browsing and those effects were generally retained across spatial scales. However, our results show there is some taxa dependent variation in the extent to which damage is reduced. Lastly, we found that most non‐lethal browsing control strategies show no significant loss of efficacy over time. Below we discuss these core findings and the implications they have for land managers.

### (1) Efficacy of non‐lethal mammal browsing control

Non‐lethal mammal browsing control significantly reduced browsing damage across all spatial scales. However, the magnitude of reduction varied between methods. The most effective measures were exclusion fencing, tree guards and repellents, which were the three most widely applied strategies. Repellents significantly reduced damage at all spatial scales, whereas exclusion fencing and tree guards reduced browsing damage at the site and landscape scale (note that the latter was only implemented at site and landscape scales). These results align with previous reviews that have demonstrated the broad effectiveness of non‐lethal controls in reducing deer browsing impacts (Guerisoli & Pereira, 2020; Redick & Jacobs, 2020). Beyond these effects, several additional trends emerged from our data. First, while tree guards and repellents generally reduced mammal browsing, there was substantial variation in this effect within each spatial scale. This likely reflects variation amongst studies in how these methods are applied. For example, both the material used and height of tree guards varied considerably, and different types of tree guard have been shown to reduce browsing to differing degrees (Thyroff *et al*., 2022). Similarly, our analysis included 85 different repellent substances, and previous research highlights that different repellents can have substantially different effects (Guerisoli & Pereira, 2020). This variation in effect was not limited to tree guards and repellents; it was common across non‐lethal control strategies.

These results are pertinent for land managers as they represent the need to tailor an approach to the browsing mammals that are present to ensure adequate damage mitigation. For example, selecting a repellent that is effective against the target species or ensuring fences are of an adequate height to prohibit access by the browsers present. Second, our meta‐analytical approach identified several other non‐lethal strategies – often absent from previous reviews – that also contributed to a significant reduction in browsing damage. For instance, natural debris performed as well as tree guards at the site scale. However, there was often insufficient data to confirm the effectiveness of these methods. This lack of data was common across several strategies, including stock manipulation, other fencing types, and neighbour effects. This was particularly true at the landscape scale, where logistical and financial barriers to large‐scale experimentation are substantial (Jenerette & Shen, 2012). Despite these challenges, we encourage further research on these underexplored approaches, especially given recent promising evidence that novel techniques, such as olfactory miscommunication (classified here as a neighbour effect), can significantly reduce mammal browsing (Finnerty *et al*., 2024).

### (2) Retention of efficacy by non‐lethal mammal browsing control methods over time

We reviewed, for the first time, the retention of efficacy of different non‐lethal mammal browsing control strategies finding subtle variation between control types. Exclusion fencing maintained its ability to reduce browsing damage for up to 10 years at site scales and up to 20 years at landscape scales. Tree guards retained their effect marginally better, albeit non‐significantly, than exclusion fencing at both site and landscape scales for up to 5 years. These results contradict concerns that tree guards lose effectiveness once trees outgrow them (Nomiya *et al*., 2022). Repellents demonstrated surprisingly durable impacts, with no significant reduction in effect relative to exclusion fencing over 5 years at the landscape scale. This durability challenges assumptions in the literature that repellents are only short‐term solutions due to issues like fresh growth not being treated or weather‐related degradation of active ingredients (Miller *et al*., 2008). It should be noted much of the existing research on repellents has been conducted over relatively short timeframes potentially overlooking longer‐term benefits. One explanation for the sustained impacts we observed here is that temporary early protection from repellents or tree guards allows seedlings to grow taller and accumulate greater biomass, making them more resistant to subsequent browsing damage (Welch *et al*., 1991). This can lead to a sustained benefit beyond the initial window of protection. In reference to the longevity of repellents, it should be noted that our meta‐analysis could not separate studies where repellents were periodically reapplied (e.g. Swihart, Pignatello & Mattina, 1991), which may prolong effectiveness by protecting new growth, thus presenting a more long‐lived effect than a single application. Further investigation surrounding the longevity of effect gained from repeat application of repellents would allow for better informed deployment of repellent‐based mammal browsing control.

### (3) Influence of browsing taxa on efficacy of non‐lethal mammal browsing control

At all spatial scales there was variation in the level of reduction between different mammal groups. Browsing by deer was significantly reduced by non‐lethal control at both site and landscape scale. Similarly, browsing by other ungulates (e.g. elk, moose and reindeer) was significantly reduced at all scales. The similarities between these effects may be due to similar morphologies, browsing behaviours and overlapping forage preferences between deer and other ungulates (Crête, Ouellet and Lesage, 2001). At the site scale browsing by other and mixed mammal assemblages was reduced to a similar extent to the ungulate groups but at the landscape scale the reduction in browsing by those groups was lesser. It is not clear why these effects differ across spatial scales but it is possible that the variation in types of browsing (e.g. bark stripping versus foliage removal or uprooting) within the more diverse other and mixed mammal categories require more complex browsing control strategies to mitigate damage effectively. This complexity may be achievable at a site scale, but at a landscape scale it may become too costly potentially explaining the reduced effectiveness.

### (4) Practical considerations

Use of lethal control can be socially polarising (Boulet *et al*., 2021). Given non‐lethal approaches can be as efficient, in some circumstances, as lethal control (Fig. 2 in Redick & Jacobs, 2020) why are non‐lethal strategies not the predominant practice for controlling browsing mammals? Balancing the input cost of one strategy versus another while incorporating the longevity and efficacy of those strategies is not straight forward. Moreover, there are often other practical considerations. For example, we demonstrated that tree guarding significantly reduces browsing damage for long periods at the site and landscape scales, but they can have negative environmental impacts. Guards are often made of plastic that remains in the environment after its protection is no longer necessary and collection and reuse of guards, while possible, is often impractical at large scales (Scott, 2023). While biodegradable alternatives do exist, the height of trees is significantly less than those grown in plastic guards (Brown *et al*., 2018). Other control measures have similar issues. Exclusion fencing, while providing effective, long‐lasting protection, can be untenably expensive if ground is steep (Forsyth, 2023) or logistically challenging given forestry land is often crossed by public roads. Lastly, land managers are often managing multiple species with different browsing behaviours. Co‐existing mammals that cause different types of browsing damage have been shown to be better combatted by a combination of different non‐lethal strategies (Miller *et al*., 2009, 2011). Applying multiple treatments, especially at large spatial scales, can dramatically increase the cost of control operations. Land managers must, therefore, balance these competing factors when selecting a browsing control strategy if their goals of timber production, reforestation, or otherwise are to be met. It is also vital that there is continued investment into the research and development of non‐lethal mammal browsing control strategies – be that environmentally friendly tree guards or cheaper exclusion fencing strategies – so the challenges facing non‐lethal control can be lessened and non‐lethal control be made more accessible.

## V. CONCLUSIONS

(1) Non‐lethal mammal browsing control strategies significantly reduce browsing damage across spatial scales and mammal taxa.
(2) The effects of non‐lethal browsing control can be retained for considerable periods of time.
(3) There is substantial variation within strategies at different scales, but non‐lethal browsing control was on average highly effective and our results provide land managers with confidence in the efficacy of several alternative strategies that could replace or be used in conjunction with reduced lethal measures.
(4) There are other factors that need to be considered when selecting a non‐lethal browsing control strategy – our results provide a strong foundation for land managers in making useful, proactive, and effective decisions.

## AUTHOR CONTRIBUTIONS

J.B.H.G., G.M.W., D.W., J.O.W. conceived the study, J.B.H.G. performed the literature search and data extraction, J.B.H.G. and D.W.A.N. processed the data and conducted the meta‐analyses. J.B.H.G. wrote the first draft of the manuscript and all authors contributed substantially to revisions.

## Supporting information


**Appendix S1.** Missing data.
**Figure S1.** PRISMA (preferred reporting items for systematic reviews and meta‐analyses) flow diagram of the literature search conducted for this meta‐analysis.
**Figure S2.** Experiments with shared controls and those that tested combinations of two treatments.
**Figure S3.** Orchard plots showing total effect sizes (*k*) across *n* studies, in brackets, for non‐lethal mammal browsing control at a ‘site scale’.
**Figure S4.** Bubble plots showing the retention of efficacy over time for different non‐lethal mammal browsing control methods at a ‘site scale’.
**Table S1.** Plant scale multilevel meta‐analytic model (MLMA).
**Table S2.** Plant scale MLMA publication bias. root_inv_ESS = effective sample size.
**Table S3.** Plant scale Multilevel Meta‐regression (MLMR) – suppressed intercept, control method.
**Table S4.** Plant scale MLMR– suppressed intercept, control method publication bias.
**Table S5.** Plant scale MLMR – suppressed intercept, browsing taxa.
**Table S6.** Plant scale MLMR – suppressed intercept, browsing taxa publication bias.
**Table S7.** Plant scale MLMR – control method efficacy through time (repellents only).
**Table S8.** Plant scale MLMR – control method efficacy through time publication bias.
**Table S9.** Site scale Multilevel MLMA.
**Table S10.** Site scale MLMA publication bias.
**Table S11.** Site scale MLMR – suppressed intercept, control method.
**Table S12.** Site scale MLMR – suppressed intercept, control method publication bias.
**Table S13.** Site scale MLMR – suppressed intercept, browsing taxa.
**Table S14.** Site scale MLMR – suppressed intercept, browsing taxa publication bias.
**Table S15.** Site scale MLMR – control method efficacy through time.
**Table S16.** Site scale MLMR – control method efficacy through time publication bias.
**Table S17.** Landscape scale MLMA.
**Table S18.** Landscape scale MLMA publication bias.
**Table S19.** Landscape scale MLMR – suppressed intercept, control method.
**Table S20.** Landscape scale MLMR – suppressed intercept, control method publication bias.
**Table S21.** Landscape scale MLMR – suppressed intercept, browsing taxa.
**Table S22.** Landscape scale MLMR – suppressed intercept, browsing taxa publication bias.
**Table S23.** Landscape scale MLMR – control method efficacy through time.
**Table S24.** Landscape scale MLMR – control method efficacy through time publication bias.
**Table S25.** Plant scale MLMA heterogeneity.
**Table S26.** Site scale MLMA heterogeneity.
**Table S27.** Landscape scale MLMA heterogeneity.
**Appendix S2.** Meta‐analyses reference list.

## Data Availability

All data and annotated code are provided in ‘Browse_meta’ at https://github.com/jbhg97/Browse_meta or https://doi.org/10.5281/zenodo.19937784.
