# Supplementary material for: A global meta‐analysis of non‐lethal mammal browsing control in forests
Source: Biol Rev Camb Philos Soc. 2026 Aug 23;101(6):2901–19. doi: 10.1002/brv.70205 (PMC13736800; doi:10.1002/brv.70205)
Supplement: Supplementary file 1 [file BRV-101-2901-s001.docx]

**Supporting Information: A global meta-analysis of non-lethal mammal browsing control in forests**

James B.H. Grimsdale, Daniel W.A. Noble, Geoffrey M. While, Dean Williams and Julianne O’Reilly-Wapstra

**Appendix S1.**

**Missing Data**

Multivariate imputation with 20 iterations was carried out using predictive mean matching in the R package *mice*, (version 3.16.0; van Buuren & Groothuis-Oudshoorn, 2011). A predictor matrix was used. For imputation of missing standard deviations, Paper ID, plant genus, scale, plot size, time, sample size, mean measurement and standard deviation were used. For imputing missing plot size values, we used Paper ID, scale and sample size. Imputation of missing data allows for greater numbers of effects, increasing the power of a meta-analysis. However, imputation comes with a level of uncertainty. Therefore, we conducted a complete-case meta-analysis and analysed the dataset containing imputed effects as a sensitivity analysis, as advocated by Kambach *et al*. (2020). Code detailing the imputation process can be found in the Meta_prelim_process.qmd within the Data processing folder of the Browse_meta github repository identified in our data accessibility statement (<https://github.com/jbhg97/Browse_meta> or <https://doi.org/10.5281/zenodo.17084163>).

**Supplementary References**

Kambach, S., Bruelheide, H., Gerstner, K., Gurevitch, J., Beckmann, M. & Seppelt, R. (2020). Consequences of multiple imputation of missing standard deviations and sample sizes in meta‐analysis. *Ecology and Evolution* **10**, 11699–11712.

Van Buuren, S. & Groothuis-Oudshoorn, K. (2011). Mice: multivariate imputation by chained equations in R. *Journal of statistical software* **45**, 1–67.

**Fig. S1.** PRISMA (preferred reporting items for systematic reviews and meta-analyses) flow diagram of the literature search conducted for this meta-analysis. The template for this diagram came from Page, M.J. *et al*. (2021). The PRISMA, 2020 statement: an updated guideline for reporting systematic reviews. *Systematic Reviews*, **10**, 89.

Records identified from:

Web of Science (n = 397)

Scopus (n = 118)

Records removed *before screening*:

Duplicate records removed

(n = 95)

Records removed for other reasons (n = 0)

Records screened

(n = 420)

Records excluded

(n = 196)

Reports sought for retrieval

(n = 224)

Reports not retrieved

(n = 0)

Reports assessed for eligibility

(n = 224)

Reports excluded:

Selection criteria (n = 68)

Data unavailable from authors (n = 23)

Records identified from:

Review reference lists (n = 266)

Reports assessed for eligibility

(n = 28)

Reports excluded:

Data unavailable from authors (n = 5)

Studies included in review

(n = 156; 112 complete-case analysis/156 imputed-case analysis)

**Identification of studies via databases and registers**

**Identification of studies via other methods**

**Identification**

**Screening**

**Included**

Reports sought for retrieval

(n = 28)

Reports not retrieved

(n = 0)

Records excluded

Duplicates or title and abstract screening (n = 238)

A

B

**Fig. S2.** Experiments with shared controls (A) were common. As were those that tested combinations of two treatments (B). In both instances either control or treatment data is shared across multiple effect sizes creating non-independence (see inset tables). This was accounted for using variance-covariance matrices with *r* = 0.5.

**Fig. S3.** Orchard plots showing total effect sizes (k) across *n* studies, in brackets, for non-lethal mammal browsing control at a ‘site scale’. Bubbles represent individual effect sizes, increasing size denotes increasing precision; positive effect size represents a reduction in browsing damage. Points show marginalised mean effect size for different control methods (A) and different browsing taxa (B). Thick lines show 95% confidence intervals and thin lines show 95% prediction intervals. SMD = standardised mean difference.

A

**Fig. S4.** Bubble plots showing the retention of efficacy over time for different non-lethal mammal browsing control methods at a ‘site scale’. Panel A shows exclusion fencing, repellents, tree guards and other fencing; B shows natural debris, stock manipulation and neighbour effects. Methods and number of effect sizes (k) are shown in subplot titles. The size of each bubble denotes the effect size precision (see subplot legends). Solid lines denote the marginalised mean effect size. Dashed lines show the 95% confidence intervals and dotted lines show the 95% prediction intervals. Note the different subplot legends and different scales of the X and Y axes. SMD = standardised mean difference.

B

## **Table S1.** Plant scale multilevel meta-analytic model (MLMA). est = estimate (mean effect) ; ci.lb = confidence interval lower bound; ci.ub = confidence interval upper bound; se = standard error; stat = Z test score; p.val = *P* value.

|  | Complete case | | | | | | Robust | | | | Imputed case | | | |
| --- | --- | --- | --- | --- | --- | --- | --- | --- | --- | --- | --- | --- | --- | --- |
| term | est | ci.lb | ci.ub | se | stat | p.val | est | se | stat | p.val | est | se | stat | p.val |
| intrcpt | 1.02424 | 0.47114 | 1.57734 | 0.23985 | 4.27028 | 0.00272 | 1.02424 | 0.23904 | 4.28487 | 0.00129 | 1.19212 | 0.25503 | 4.67446 | 0.00001 |

## **Table S2.** Plant scale MLMA publication bias. root_inv_ESS = effective sample size.

|  | Complete case | | | | | | Robust | | | | | |
| --- | --- | --- | --- | --- | --- | --- | --- | --- | --- | --- | --- | --- |
| term | est | ci.lb | ci.ub | se | stat | p.val | est | ci.lb | ci.ub | se | stat | p.val |
| intrcpt | 0.93628 | -0.02229 | 1.89486 | 0.39175 | 2.39000 | 0.05403 | 0.93628 | 0.14051 | 1.73205 | 0.35177 | 2.66160 | 0.02598 |
| Year | -0.02285 | -0.06730 | 0.02161 | 0.01965 | -1.16267 | 0.27487 | -0.02285 | -0.05720 | 0.01151 | 0.01519 | -1.50439 | 0.16674 |
| root_inv_ESS | 0.50026 | -1.21763 | 2.21814 | 0.86869 | 0.57588 | 0.56565 | 0.50026 | -0.34029 | 1.34080 | 0.37157 | 1.34633 | 0.21112 |

## **Table S3.** Plant scale Multilevel Meta-regression (MLMR) – suppressed intercept, control method.

|  | Complete case | | | | | | Robust | | | | Imputed case | | | |
| --- | --- | --- | --- | --- | --- | --- | --- | --- | --- | --- | --- | --- | --- | --- |
| term | est | ci.lb | ci.ub | se | stat | p.val | est | se | stat | p.val | est | se | stat | p.val |
| Exclusion fence | 0.27812 | -3.70651 | 4.26274 | 1.62843 | 0.17079 | 0.87000 | 0.27812 | 0.73226 | 0.37981 | 0.71290 | 0.33283 | 2.41216 | 0.13798 | 0.89075 |
| Repellents | 0.83088 | 0.13794 | 1.52383 | 0.28319 | 2.93399 | 0.02615 | 0.83088 | 0.30060 | 2.76409 | 0.02196 | 1.08907 | 0.32393 | 3.36203 | 0.00105 |
| Plot_size | 0.00011 | -0.00012 | 0.00035 | 0.00012 | 0.95749 | 0.34092 | 0.00011 | 0.00006 | 1.87173 | 0.09404 | 0.00011 | 0.00018 | 0.61095 | 0.54450 |

## **Table S4.** Plant scale MLMR – suppressed intercept, control method publication bias.

|  | Complete case | | | | | | Robust | | | | | |
| --- | --- | --- | --- | --- | --- | --- | --- | --- | --- | --- | --- | --- |
| term | est | ci.lb | ci.ub | se | stat | p.val | est | ci.lb | ci.ub | se | stat | p.val |
| Exclusion fence | 0.23787 | -4.53061 | 5.00635 | 1.71748 | 0.13850 | 0.89654 | 0.23787 | -2.81836 | 3.29410 | 1.29248 | 0.18404 | 0.85920 |
| Repellents | 0.65492 | -0.60068 | 1.91053 | 0.45224 | 1.44819 | 0.22114 | 0.65492 | -0.65258 | 1.96242 | 0.55294 | 1.18443 | 0.27489 |
| Plot_size | 0.00011 | -0.00014 | 0.00035 | 0.00012 | 0.86447 | 0.38971 | 0.00011 | -0.00012 | 0.00033 | 0.00010 | 1.09426 | 0.31006 |
| Year | -0.01941 | -0.06664 | 0.02781 | 0.01997 | -0.97208 | 0.36339 | -0.01941 | -0.06926 | 0.03044 | 0.02108 | -0.92090 | 0.38774 |
| root_inv_ESS | 0.73460 | -1.03071 | 2.49992 | 0.89255 | 0.82303 | 0.41195 | 0.73460 | -0.50674 | 1.97595 | 0.52497 | 1.39933 | 0.20443 |

## **Table S5.** Plant scale MLMR – suppressed intercept, browsing taxa.

|  | Complete case | | | | | | Robust | | | | Imputed case | | | |
| --- | --- | --- | --- | --- | --- | --- | --- | --- | --- | --- | --- | --- | --- | --- |
| term | est | ci.lb | ci.ub | se | stat | p.val | est | se | stat | p.val | est | se | stat | p.val |
| Deer | 0.87274 | -0.02738 | 1.77286 | 0.45294 | 1.92685 | 0.05723 | 0.87274 | 0.29941 | 2.91486 | 0.01944 | 0.83806 | 0.50048 | 1.67452 | 0.09620 |
| Other | 0.73476 | -0.09072 | 1.56024 | 0.35797 | 2.05257 | 0.07421 | 0.73476 | 0.50002 | 1.46944 | 0.17991 | 1.22795 | 0.40838 | 3.00688 | 0.00311 |
| Other ungulates | 1.65403 | 0.34747 | 2.96059 | 0.65746 | 2.51580 | 0.01369 | 1.65403 | 0.44978 | 3.67744 | 0.00624 | 1.26293 | 0.82909 | 1.52327 | 0.13200 |
| Plot_size | -0.00000 | -0.00017 | 0.00017 | 0.00009 | -0.01774 | 0.98589 | -0.00000 | 0.00004 | -0.03386 | 0.97382 | 0.00005 | 0.00014 | 0.36537 | 0.71704 |

## **Table S6.** Plant scale MLMR – suppressed intercept, browsing taxa publication bias.

|  | Complete case | | | | | | Robust | | | | | |
| --- | --- | --- | --- | --- | --- | --- | --- | --- | --- | --- | --- | --- |
| term | est | ci.lb | ci.ub | se | stat | p.val | est | ci.lb | ci.ub | se | stat | p.val |
| Deer | 1.18171 | -0.09779 | 2.46121 | 0.64363 | 1.83600 | 0.06981 | 1.18171 | -0.14183 | 2.50525 | 0.54090 | 2.18470 | 0.07160 |
| Other | 0.58941 | -0.49729 | 1.67611 | 0.44411 | 1.32716 | 0.23272 | 0.58941 | -0.89067 | 2.06948 | 0.60487 | 0.97443 | 0.36748 |
| Other ungulates | 2.11236 | 0.33725 | 3.88746 | 0.89294 | 2.36563 | 0.02025 | 2.11236 | 0.21527 | 4.00945 | 0.77530 | 2.72457 | 0.03443 |
| Plot_size | -0.00005 | -0.00024 | 0.00013 | 0.00009 | -0.57481 | 0.56692 | -0.00005 | -0.00024 | 0.00013 | 0.00007 | -0.72594 | 0.49521 |
| Year | -0.03896 | -0.09301 | 0.01509 | 0.02209 | -1.76378 | 0.12822 | -0.03896 | -0.09237 | 0.01444 | 0.02183 | -1.78515 | 0.12449 |
| root_inv_ESS | 0.35053 | -1.43209 | 2.13315 | 0.90124 | 0.38894 | 0.69794 | 0.35053 | -1.18851 | 1.88957 | 0.62897 | 0.55730 | 0.59748 |

## **Table S7.** Plant scale MLMR – control method efficacy through time (repellents only).

|  | Complete case | | | | | | Robust | | | | Imputed case | | | |
| --- | --- | --- | --- | --- | --- | --- | --- | --- | --- | --- | --- | --- | --- | --- |
| term | est | ci.lb | ci.ub | se | stat | p.val | est | se | stat | p.val | est | se | stat | p.val |
| intrcpt | 0.86956 | -0.00227 | 1.74138 | 0.31401 | 2.76923 | 0.05037 | 0.86956 | 0.31500 | 2.76049 | 0.02808 | 1.15814 | 0.35894 | 3.22660 | 0.00166 |
| Months | -0.47308 | -1.63217 | 0.68600 | 0.58532 | -0.80825 | 0.42057 | -0.47308 | 0.26460 | -1.78794 | 0.11693 | -0.55905 | 0.66545 | -0.84011 | 0.40228 |
| Plot_size | 0.00012 | -0.00013 | 0.00036 | 0.00012 | 0.95358 | 0.34321 | 0.00012 | 0.00006 | 1.88774 | 0.10101 | 0.00011 | 0.00019 | 0.61022 | 0.54504 |

## **Table S8.** Plant scale MLMR – control method efficacy through time publication bias.

|  | Complete case | | | | | | Robust | | | | | |
| --- | --- | --- | --- | --- | --- | --- | --- | --- | --- | --- | --- | --- |
| term | est | ci.lb | ci.ub | se | stat | p.val | est | ci.lb | ci.ub | se | stat | p.val |
| intrcpt | 0.80407 | -1.20492 | 2.81306 | 0.46692 | 1.72208 | 0.22720 | 0.80407 | -0.62962 | 2.23776 | 0.55773 | 1.44169 | 0.20896 |
| Months | -0.55415 | -1.72620 | 0.61790 | 0.59176 | -0.93645 | 0.35099 | -0.55415 | -1.37779 | 0.26949 | 0.32041 | -1.72951 | 0.14428 |
| Plot_size | 0.00010 | -0.00015 | 0.00035 | 0.00013 | 0.78613 | 0.43420 | 0.00010 | -0.00016 | 0.00036 | 0.00010 | 0.97546 | 0.37413 |
| Year | -0.02361 | -0.08013 | 0.03290 | 0.02199 | -1.07396 | 0.33191 | -0.02361 | -0.08511 | 0.03788 | 0.02392 | -0.98697 | 0.36898 |
| root_inv_ESS | 0.65990 | -1.22022 | 2.54002 | 0.94419 | 0.69891 | 0.48671 | 0.65990 | -0.42906 | 1.74887 | 0.42363 | 1.55775 | 0.18003 |

## **Table S9.** Site scale Multilevel MLMA.

|  | Complete case | | | | | | Robust | | | | Imputed case | | | |
| --- | --- | --- | --- | --- | --- | --- | --- | --- | --- | --- | --- | --- | --- | --- |
| term | est | ci.lb | ci.ub | se | stat | p.val | est | se | stat | p.val | est | se | stat | p.val |
| intrcpt | 0.64098 | 0.32761 | 0.95435 | 0.15365 | 4.17170 | 0.00023 | 0.64098 | 0.11939 | 5.36873 | 0.00000 | 0.63389 | 0.13117 | 4.83269 | 0.00000 |

## **Table S10.** Site scale MLMA publication bias.

|  | Complete case | | | | | | Robust | | | | | |
| --- | --- | --- | --- | --- | --- | --- | --- | --- | --- | --- | --- | --- |
| term | est | ci.lb | ci.ub | se | stat | p.val | est | ci.lb | ci.ub | se | stat | p.val |
| intrcpt | 0.71822 | 0.33961 | 1.09682 | 0.18512 | 3.87982 | 0.00055 | 0.71822 | 0.35315 | 1.08329 | 0.18090 | 3.97029 | 0.00028 |
| Year | 0.00471 | -0.01357 | 0.02299 | 0.00906 | 0.51993 | 0.60584 | 0.00471 | -0.00809 | 0.01751 | 0.00634 | 0.74277 | 0.46176 |
| root_inv_ESS | -0.13978 | -0.62110 | 0.34154 | 0.24532 | -0.56979 | 0.56893 | -0.13978 | -0.58548 | 0.30591 | 0.22085 | -0.63292 | 0.53022 |

## **Table S11.** Site scale MLMR – suppressed intercept, control method.

|  | Complete case | | | | | | Robust | | | | Imputed case | | | |
| --- | --- | --- | --- | --- | --- | --- | --- | --- | --- | --- | --- | --- | --- | --- |
| term | est | ci.lb | ci.ub | se | stat | p.val | est | se | stat | p.val | est | se | stat | p.val |
| Exclusion fence | 0.59514 | 0.20204 | 0.98825 | 0.20009 | 2.97437 | 0.00308 | 0.59514 | 0.18544 | 3.20937 | 0.00275 | 0.60201 | 0.17161 | 3.50796 | 0.00046 |
| Natural debris | 1.20491 | 0.35846 | 2.05136 | 0.43084 | 2.79666 | 0.00536 | 1.20491 | 0.49548 | 2.43183 | 0.01998 | 0.78483 | 0.31746 | 2.47219 | 0.01360 |
| Neighbour effects | -0.23324 | -1.00230 | 0.53582 | 0.39145 | -0.59584 | 0.55155 | -0.23324 | 0.16973 | -1.37418 | 0.17765 | -0.29653 | 0.38197 | -0.77632 | 0.43767 |
| Other fencing | 0.72799 | -0.11561 | 1.57159 | 0.42939 | 1.69542 | 0.09061 | 0.72799 | 0.24541 | 2.96638 | 0.00525 | 0.61780 | 0.38310 | 1.61267 | 0.10700 |
| Repellents | 0.56294 | 0.14204 | 0.98385 | 0.21424 | 2.62767 | 0.00886 | 0.56294 | 0.26291 | 2.14121 | 0.03891 | 0.51736 | 0.16532 | 3.12950 | 0.00179 |
| Stock manipulation | 0.22735 | -0.40559 | 0.86029 | 0.32216 | 0.70569 | 0.48070 | 0.22735 | 0.21139 | 1.07552 | 0.28911 | 0.34340 | 0.27052 | 1.26943 | 0.20456 |
| Tree guards | 1.20081 | 0.76946 | 1.63217 | 0.21956 | 5.46924 | 0.00000 | 1.20081 | 0.23280 | 5.15814 | 0.00001 | 1.22203 | 0.17663 | 6.91849 | 0.00000 |
| Plot_size | -0.00001 | -0.00005 | 0.00004 | 0.00002 | -0.27580 | 0.78281 | -0.00001 | 0.00001 | -0.89290 | 0.37768 | -0.00001 | 0.00002 | -0.26824 | 0.78858 |

## **Table S12.** Site scale MLMR – suppressed intercept, control method publication bias.

|  | Complete case | | | | | | Robust | | | | | |
| --- | --- | --- | --- | --- | --- | --- | --- | --- | --- | --- | --- | --- |
| term | est | ci.lb | ci.ub | se | stat | p.val | est | ci.lb | ci.ub | se | stat | p.val |
| Exclusion fence | 0.67090 | 0.23709 | 1.10471 | 0.22081 | 3.03842 | 0.00250 | 0.67090 | 0.20417 | 1.13763 | 0.22991 | 2.91816 | 0.00612 |
| Natural debris | 1.35364 | 0.42965 | 2.27763 | 0.47030 | 2.87823 | 0.00417 | 1.35364 | 0.60818 | 2.09910 | 0.36720 | 3.68634 | 0.00077 |
| Neighbour effects | -0.15439 | -0.94914 | 0.64036 | 0.40452 | -0.38167 | 0.70286 | -0.15439 | -0.59217 | 0.28338 | 0.21564 | -0.71597 | 0.47876 |
| Other fencing | 0.82895 | -0.05168 | 1.70959 | 0.44824 | 1.84937 | 0.06499 | 0.82895 | 0.15496 | 1.50295 | 0.33200 | 2.49685 | 0.01739 |
| Repellents | 0.67087 | 0.18015 | 1.16159 | 0.24977 | 2.68593 | 0.00747 | 0.67087 | -0.03779 | 1.37953 | 0.34907 | 1.92186 | 0.06280 |
| Stock manipulation | 0.30097 | -0.35414 | 0.95608 | 0.33344 | 0.90261 | 0.36717 | 0.30097 | -0.19393 | 0.79587 | 0.24378 | 1.23458 | 0.22521 |
| Tree guards | 1.30138 | 0.81225 | 1.79051 | 0.24896 | 5.22719 | 0.00000 | 1.30138 | 0.68320 | 1.91956 | 0.30451 | 4.27375 | 0.00014 |
| Plot_size | -0.00001 | -0.00005 | 0.00004 | 0.00002 | -0.22253 | 0.82399 | -0.00001 | -0.00002 | 0.00001 | 0.00001 | -0.69889 | 0.48924 |
| Year | 0.00666 | -0.01469 | 0.02802 | 0.01052 | 0.63354 | 0.53050 | 0.00666 | -0.01457 | 0.02790 | 0.01046 | 0.63695 | 0.52830 |
| root_inv_ESS | -0.13396 | -0.59389 | 0.32597 | 0.23442 | -0.57147 | 0.56780 | -0.13396 | -0.64794 | 0.38002 | 0.25318 | -0.52912 | 0.60006 |

## **Table S13.** Site scale MLMR – suppressed intercept, browsing taxa.

|  | Complete case | | | | | | Robust | | | | Imputed case | | | |
| --- | --- | --- | --- | --- | --- | --- | --- | --- | --- | --- | --- | --- | --- | --- |
| term | est | ci.lb | ci.ub | se | stat | p.val | est | se | stat | p.val | est | se | stat | p.val |
| Deer | 0.63223 | 0.20353 | 1.06092 | 0.21850 | 2.89352 | 0.00388 | 0.63223 | 0.17451 | 3.62284 | 0.00083 | 0.69763 | 0.17882 | 3.90137 | 0.00010 |
| Mixed | 0.63087 | -0.25267 | 1.51440 | 0.45032 | 1.40093 | 0.16150 | 0.63087 | 0.26680 | 2.36454 | 0.02313 | 0.49044 | 0.45154 | 1.08615 | 0.27756 |
| Not specified | 0.61615 | -0.05552 | 1.28783 | 0.33207 | 1.85550 | 0.07109 | 0.61615 | 0.26668 | 2.31043 | 0.02624 | 0.70463 | 0.35344 | 1.99365 | 0.04634 |
| Other | 0.87929 | 0.10470 | 1.65388 | 0.38295 | 2.29609 | 0.02713 | 0.87929 | 0.32902 | 2.67246 | 0.01093 | 0.73532 | 0.34842 | 2.11043 | 0.03496 |
| Other ungulates | 0.61298 | 0.03253 | 1.19344 | 0.28697 | 2.13605 | 0.03901 | 0.61298 | 0.38111 | 1.60844 | 0.11581 | 0.49882 | 0.24092 | 2.07049 | 0.03856 |
| Plot_size | -0.00001 | -0.00006 | 0.00003 | 0.00002 | -0.61338 | 0.53990 | -0.00001 | 0.00001 | -1.23282 | 0.22502 | -0.00001 | 0.00002 | -0.52917 | 0.59682 |

## **Table S14.** Site scale MLMR – suppressed intercept, browsing taxa publication bias.

|  | Complete case | | | | | | Robust | | | | | |
| --- | --- | --- | --- | --- | --- | --- | --- | --- | --- | --- | --- | --- |
| term | est | ci.lb | ci.ub | se | stat | p.val | est | ci.lb | ci.ub | se | stat | p.val |
| Deer | 0.73907 | 0.24091 | 1.23723 | 0.25390 | 2.91082 | 0.00367 | 0.73907 | 0.32792 | 1.15022 | 0.20292 | 3.64219 | 0.00082 |
| Mixed | 0.73295 | -0.19372 | 1.65963 | 0.47231 | 1.55186 | 0.12097 | 0.73295 | 0.08040 | 1.38551 | 0.32206 | 2.27585 | 0.02874 |
| Not specified | 0.66816 | -0.02511 | 1.36143 | 0.34215 | 1.95280 | 0.05844 | 0.66816 | 0.09626 | 1.24005 | 0.28225 | 2.36725 | 0.02326 |
| Other | 1.01254 | 0.15829 | 1.86680 | 0.42161 | 2.40163 | 0.02146 | 1.01254 | 0.18873 | 1.83636 | 0.40658 | 2.49037 | 0.01738 |
| Other ungulates | 0.67061 | 0.06283 | 1.27839 | 0.29996 | 2.23567 | 0.03149 | 0.67061 | -0.18033 | 1.52156 | 0.41997 | 1.59681 | 0.11881 |
| Plot_size | -0.00001 | -0.00006 | 0.00003 | 0.00002 | -0.55554 | 0.57877 | -0.00001 | -0.00004 | 0.00001 | 0.00001 | -1.05434 | 0.29856 |
| Year | 0.00623 | -0.01426 | 0.02672 | 0.01011 | 0.61582 | 0.54179 | 0.00623 | -0.00724 | 0.01969 | 0.00665 | 0.93691 | 0.35488 |
| root_inv_ESS | -0.14472 | -0.63171 | 0.34228 | 0.24821 | -0.58304 | 0.55998 | -0.14472 | -0.61754 | 0.32811 | 0.23336 | -0.62016 | 0.53896 |

## **Table S15.** Site scale MLMR – control method efficacy through time.

|  | Complete case | | | | | | Robust | | | | Imputed case | | | |
| --- | --- | --- | --- | --- | --- | --- | --- | --- | --- | --- | --- | --- | --- | --- |
| term | est | ci.lb | ci.ub | se | stat | p.val | est | se | stat | p.val | est | se | stat | p.val |
| intrcpt | 0.42731 | -0.14156 | 0.99617 | 0.26963 | 1.58479 | 0.13144 | 0.42731 | 0.33346 | 1.28142 | 0.21056 | 0.55044 | 0.22957 | 2.39770 | 0.01663 |
| Natural debris | 0.94532 | -0.13305 | 2.02369 | 0.54882 | 1.72245 | 0.08563 | 0.94532 | 0.67348 | 1.40363 | 0.17142 | 0.23776 | 0.37886 | 0.62757 | 0.53044 |
| Neighbour effects | -1.02417 | -1.94727 | -0.10107 | 0.46980 | -2.18003 | 0.02974 | -1.02417 | 0.08930 | -11.46935 | 0.00000 | -1.10177 | 0.49869 | -2.20932 | 0.02728 |
| Other fencing | 1.04874 | -0.51037 | 2.60785 | 0.79349 | 1.32169 | 0.18690 | 1.04874 | 0.60417 | 1.73584 | 0.09359 | 0.75937 | 0.78601 | 0.96612 | 0.33412 |
| Repellents | 0.24365 | -0.31891 | 0.80622 | 0.28631 | 0.85100 | 0.39519 | 0.24365 | 0.54018 | 0.45105 | 0.65542 | 0.05294 | 0.25211 | 0.20998 | 0.83371 |
| Stock manipulation | -0.34685 | -1.10360 | 0.40991 | 0.38514 | -0.90057 | 0.36826 | -0.34685 | 0.41787 | -0.83003 | 0.41354 | -0.51221 | 0.34221 | -1.49678 | 0.13494 |
| Tree guards | 0.38513 | -0.18157 | 0.95183 | 0.28841 | 1.33535 | 0.18239 | 0.38513 | 0.48809 | 0.78907 | 0.43670 | 0.39472 | 0.25927 | 1.52247 | 0.12813 |
| Months | 0.00252 | -0.00326 | 0.00831 | 0.00295 | 0.85688 | 0.39169 | 0.00252 | 0.00326 | 0.77500 | 0.44484 | 0.00122 | 0.00309 | 0.39339 | 0.69411 |
| Plot_size | -0.00001 | -0.00005 | 0.00004 | 0.00002 | -0.24984 | 0.80282 | -0.00001 | 0.00001 | -0.88140 | 0.38560 | -0.00001 | 0.00002 | -0.28853 | 0.77300 |
| Natural debris:Months | -0.01056 | -0.03275 | 0.01162 | 0.01131 | -0.93426 | 0.35037 | -0.01056 | 0.00383 | -2.75812 | 0.01012 | -0.00218 | 0.01044 | -0.20840 | 0.83504 |
| Neighbour effects:Months | -0.00277 | -0.02793 | 0.02240 | 0.01283 | -0.21562 | 0.82933 | -0.00277 | 0.00205 | -1.34862 | 0.18827 | -0.00228 | 0.01640 | -0.13934 | 0.88920 |
| Other fencing:Months | -0.08379 | -0.19966 | 0.03208 | 0.05906 | -1.41882 | 0.15623 | -0.08379 | 0.05027 | -1.66684 | 0.10670 | -0.05397 | 0.04690 | -1.15083 | 0.24996 |
| Repellents:Months | -0.00658 | -0.01311 | -0.00005 | 0.00333 | -1.97819 | 0.04815 | -0.00658 | 0.00348 | -1.89375 | 0.06863 | -0.00409 | 0.00359 | -1.13955 | 0.25472 |
| Stock manipulation:Months | -0.00229 | -0.01412 | 0.00954 | 0.00603 | -0.37911 | 0.70467 | -0.00229 | 0.00514 | -0.44450 | 0.66010 | 0.01287 | 0.00678 | 1.90016 | 0.06003 |
| Tree guards:Months | 0.00471 | -0.00185 | 0.01127 | 0.00334 | 1.40977 | 0.15888 | 0.00471 | 0.00333 | 1.41557 | 0.16793 | 0.00649 | 0.00362 | 1.79088 | 0.07360 |

## **Table S16.** Site scale MLMR – control method efficacy through time publication bias.

|  | Complete case | | | | | | Robust | | | | | |
| --- | --- | --- | --- | --- | --- | --- | --- | --- | --- | --- | --- | --- |
| term | est | ci.lb | ci.ub | se | stat | p.val | est | ci.lb | ci.ub | se | stat | p.val |
| intrcpt | 0.50039 | -0.12089 | 1.12167 | 0.29148 | 1.71670 | 0.10661 | 0.50039 | -0.24640 | 1.24717 | 0.36331 | 1.37732 | 0.18016 |
| Natural debris | 1.03778 | -0.07249 | 2.14805 | 0.56505 | 1.83663 | 0.06688 | 1.03778 | -0.05252 | 2.12809 | 0.53043 | 1.95651 | 0.06123 |
| Neighbour effects | -1.02391 | -1.94965 | -0.09817 | 0.47114 | -2.17327 | 0.03025 | -1.02391 | -1.22462 | -0.82320 | 0.09765 | -10.48593 | 0.00000 |
| Other fencing | 1.06482 | -0.49935 | 2.62898 | 0.79605 | 1.33762 | 0.18165 | 1.06482 | -0.31834 | 2.44797 | 0.67289 | 1.58244 | 0.12564 |
| Repellents | 0.26593 | -0.30130 | 0.83317 | 0.28868 | 0.92120 | 0.35741 | 0.26593 | -0.89949 | 1.43135 | 0.56697 | 0.46904 | 0.64295 |
| Stock manipulation | -0.35735 | -1.11647 | 0.40176 | 0.38634 | -0.92498 | 0.35544 | -0.35735 | -1.24941 | 0.53470 | 0.43398 | -0.82344 | 0.41775 |
| Tree guards | 0.39931 | -0.16981 | 0.96843 | 0.28964 | 1.37864 | 0.16865 | 0.39931 | -0.64831 | 1.44693 | 0.50966 | 0.78348 | 0.44042 |
| Months | 0.00232 | -0.00350 | 0.00815 | 0.00297 | 0.78236 | 0.43417 | 0.00232 | -0.00450 | 0.00915 | 0.00332 | 0.69948 | 0.49047 |
| Plot_size | -0.00000 | -0.00005 | 0.00004 | 0.00002 | -0.18341 | 0.85455 | -0.00000 | -0.00002 | 0.00001 | 0.00001 | -0.65202 | 0.52011 |
| Year | 0.00760 | -0.01491 | 0.03011 | 0.01095 | 0.69374 | 0.49399 | 0.00760 | -0.01680 | 0.03200 | 0.01187 | 0.63999 | 0.52778 |
| root_inv_ESS | -0.04787 | -0.52737 | 0.43162 | 0.24438 | -0.19590 | 0.84473 | -0.04787 | -0.62623 | 0.53048 | 0.28137 | -0.17015 | 0.86621 |
| Natural debris:Months | -0.01086 | -0.03306 | 0.01134 | 0.01132 | -0.95976 | 0.33738 | -0.01086 | -0.01808 | -0.00364 | 0.00351 | -3.09112 | 0.00471 |
| Neighbour effects:Months | -0.00263 | -0.02779 | 0.02253 | 0.01282 | -0.20535 | 0.83733 | -0.00263 | -0.00693 | 0.00166 | 0.00209 | -1.26064 | 0.21863 |
| Other fencing:Months | -0.08354 | -0.19961 | 0.03253 | 0.05916 | -1.41216 | 0.15818 | -0.08354 | -0.19881 | 0.03174 | 0.05608 | -1.48963 | 0.14835 |
| Repellents:Months | -0.00643 | -0.01299 | 0.00012 | 0.00334 | -1.92492 | 0.05449 | -0.00643 | -0.01375 | 0.00089 | 0.00356 | -1.80675 | 0.08239 |
| Stock manipulation:Months | -0.00213 | -0.01397 | 0.00972 | 0.00604 | -0.35224 | 0.72472 | -0.00213 | -0.01293 | 0.00868 | 0.00526 | -0.40450 | 0.68915 |
| Tree guards:Months | 0.00488 | -0.00172 | 0.01148 | 0.00336 | 1.45125 | 0.14699 | 0.00488 | -0.00216 | 0.01193 | 0.00343 | 1.42399 | 0.16634 |

## **Table S17.** Landscape scale MLMA.

|  | Complete case | | | | | | Robust | | | | Imputed case | | | |
| --- | --- | --- | --- | --- | --- | --- | --- | --- | --- | --- | --- | --- | --- | --- |
| term | est | ci.lb | ci.ub | se | stat | p.val | est | se | stat | p.val | est | se | stat | p.val |
| intrcpt | 0.50833 | 0.35361 | 0.66305 | 0.07629 | 6.66328 | 0.00000 | 0.50833 | 0.07616 | 6.67439 | 0.00000 | 0.57460 | 0.08562 | 6.71099 | 0.00000 |

## **Table S18.** Landscape scale MLMA publication bias.

|  | Complete case | | | | | | Robust | | | | | |
| --- | --- | --- | --- | --- | --- | --- | --- | --- | --- | --- | --- | --- |
| term | est | ci.lb | ci.ub | se | stat | p.val | est | ci.lb | ci.ub | se | stat | p.val |
| intrcpt | 0.35098 | 0.11254 | 0.58942 | 0.11733 | 2.99145 | 0.00514 | 0.35098 | 0.11871 | 0.58325 | 0.11585 | 3.02950 | 0.00375 |
| Year | -0.00112 | -0.01696 | 0.01473 | 0.00791 | -0.14113 | 0.88829 | -0.00112 | -0.01727 | 0.01504 | 0.00806 | -0.13850 | 0.89036 |
| root_inv_ESS | 0.55462 | -0.03728 | 1.14651 | 0.30138 | 1.84024 | 0.06623 | 0.55462 | -0.18784 | 1.29708 | 0.37033 | 1.49764 | 0.14005 |

## **Table S19.** Landscape scale MLMR – suppressed intercept, control method.

|  | Complete case | | | | | | Robust | | | | Imputed case | | | |
| --- | --- | --- | --- | --- | --- | --- | --- | --- | --- | --- | --- | --- | --- | --- |
| term | est | ci.lb | ci.ub | se | stat | p.val | est | se | stat | p.val | est | se | stat | p.val |
| Exclusion fence | 0.50148 | 0.28596 | 0.71700 | 0.10963 | 4.57428 | 0.00001 | 0.50148 | 0.11793 | 4.25219 | 0.00009 | 0.55712 | 0.10946 | 5.08971 | 0.00000 |
| Natural debris | 0.30638 | -0.68746 | 1.30022 | 0.50555 | 0.60604 | 0.54483 | 0.30638 | 0.22150 | 1.38320 | 0.17287 | 0.32180 | 0.44092 | 0.72983 | 0.46580 |
| Neighbour effects | 0.54075 | -0.31558 | 1.39709 | 0.43560 | 1.24139 | 0.21518 | 0.54075 | 0.14738 | 3.66914 | 0.00060 | 0.86899 | 0.36679 | 2.36913 | 0.01818 |
| Other fencing | 0.57752 | -0.28836 | 1.44341 | 0.44046 | 1.31119 | 0.19054 | 0.57752 | 0.07080 | 8.15698 | 0.00000 | 0.54963 | 0.43073 | 1.27605 | 0.20268 |
| Repellents | 0.53586 | 0.04464 | 1.02708 | 0.24988 | 2.14450 | 0.03259 | 0.53586 | 0.26831 | 1.99715 | 0.05138 | 0.67668 | 0.22046 | 3.06947 | 0.00232 |
| Stock manipulation | -1.58859 | -2.39038 | -0.78680 | 0.40786 | -3.89497 | 0.00011 | -1.58859 | 0.71094 | -2.23448 | 0.03004 | -0.87832 | 0.42228 | -2.07993 | 0.03785 |
| Tree guards | 0.76480 | 0.29318 | 1.23641 | 0.23990 | 3.18794 | 0.00154 | 0.76480 | 0.24003 | 3.18619 | 0.00251 | 0.75548 | 0.20825 | 3.62769 | 0.00032 |
| Plot_size | 0.00011 | -0.00046 | 0.00068 | 0.00029 | 0.38589 | 0.69978 | 0.00011 | 0.00026 | 0.43397 | 0.66621 | -0.00001 | 0.00005 | -0.12336 | 0.90205 |

## **Table S20.** Landscape scale MLMR – suppressed intercept, control method publication bias.

|  | Complete case | | | | | | Robust | | | | | |
| --- | --- | --- | --- | --- | --- | --- | --- | --- | --- | --- | --- | --- |
| term | est | ci.lb | ci.ub | se | stat | p.val | est | ci.lb | ci.ub | se | stat | p.val |
| Exclusion fence | 0.32053 | 0.02533 | 0.61572 | 0.15016 | 2.13461 | 0.03340 | 0.32053 | 0.06118 | 0.57987 | 0.12892 | 2.48632 | 0.01652 |
| Natural debris | 0.21508 | -0.81454 | 1.24471 | 0.52374 | 0.41067 | 0.68154 | 0.21508 | -0.25300 | 0.68317 | 0.23268 | 0.92439 | 0.36000 |
| Neighbour effects | 0.34480 | -0.54493 | 1.23453 | 0.45258 | 0.76186 | 0.44659 | 0.34480 | -0.06291 | 0.75251 | 0.20267 | 1.70133 | 0.09549 |
| Other fencing | 0.38903 | -0.52275 | 1.30082 | 0.46380 | 0.83879 | 0.40208 | 0.38903 | 0.07980 | 0.69827 | 0.15371 | 2.53090 | 0.01478 |
| Repellents | 0.47362 | -0.06502 | 1.01226 | 0.27399 | 1.72858 | 0.08465 | 0.47362 | -0.12109 | 1.06832 | 0.29562 | 1.60213 | 0.11583 |
| Stock manipulation | -1.78099 | -2.60702 | -0.95495 | 0.42018 | -4.23862 | 0.00003 | -1.78099 | -3.39368 | -0.16830 | 0.80164 | -2.22168 | 0.03116 |
| Tree guards | 0.67852 | 0.17101 | 1.18604 | 0.25816 | 2.62831 | 0.00891 | 0.67852 | 0.19081 | 1.16624 | 0.24243 | 2.79879 | 0.00742 |
| Plot_size | 0.00006 | -0.00053 | 0.00064 | 0.00030 | 0.18476 | 0.85351 | 0.00006 | -0.00048 | 0.00059 | 0.00027 | 0.20637 | 0.83739 |
| Year | 0.00536 | -0.01359 | 0.02430 | 0.00942 | 0.56874 | 0.57224 | 0.00536 | -0.01408 | 0.02479 | 0.00966 | 0.55443 | 0.58192 |
| root_inv_ESS | 0.60211 | -0.01585 | 1.22007 | 0.31465 | 1.91361 | 0.05615 | 0.60211 | -0.18245 | 1.38667 | 0.38999 | 1.54391 | 0.12932 |

## **Table S21.** Landscape scale MLMR – suppressed intercept, browsing taxa.

|  | Complete case | | | | | | Robust | | | | Imputed case | | | |
| --- | --- | --- | --- | --- | --- | --- | --- | --- | --- | --- | --- | --- | --- | --- |
| term | est | ci.lb | ci.ub | se | stat | p.val | est | se | stat | p.val | est | se | stat | p.val |
| Deer | 0.60731 | 0.33688 | 0.87773 | 0.13470 | 4.50858 | 0.00004 | 0.60731 | 0.14538 | 4.17726 | 0.00012 | 0.72430 | 0.14770 | 4.90377 | 0.00000 |
| Mixed | 0.27920 | -0.10519 | 0.66359 | 0.19554 | 1.42788 | 0.15410 | 0.27920 | 0.08204 | 3.40306 | 0.00130 | 0.38613 | 0.22978 | 1.68040 | 0.09383 |
| Not specified | 0.78870 | 0.35964 | 1.21776 | 0.21372 | 3.69031 | 0.00055 | 0.78870 | 0.37638 | 2.09548 | 0.04111 | 0.81821 | 0.22956 | 3.56422 | 0.00039 |
| Other | 0.20067 | -0.37787 | 0.77920 | 0.28817 | 0.69634 | 0.48938 | 0.20067 | 0.27645 | 0.72586 | 0.47124 | 0.43705 | 0.28183 | 1.55078 | 0.12149 |
| Other ungulates | 0.31847 | 0.07141 | 0.56553 | 0.12568 | 2.53404 | 0.01165 | 0.31847 | 0.07675 | 4.14972 | 0.00013 | 0.42035 | 0.12981 | 3.23809 | 0.00125 |
| Plot_size | 0.00023 | -0.00028 | 0.00074 | 0.00026 | 0.89967 | 0.36883 | 0.00023 | 0.00023 | 1.01625 | 0.31431 | -0.00001 | 0.00005 | -0.23108 | 0.81773 |

## **Table S22.** Landscape scale MLMR – suppressed intercept, browsing taxa publication bias.

|  | Complete case | | | | | | Robust | | | | | |
| --- | --- | --- | --- | --- | --- | --- | --- | --- | --- | --- | --- | --- |
| term | est | ci.lb | ci.ub | se | stat | p.val | est | ci.lb | ci.ub | se | stat | p.val |
| Deer | 0.45918 | 0.14149 | 0.77687 | 0.15809 | 2.90460 | 0.00550 | 0.45918 | 0.12012 | 0.79825 | 0.16872 | 2.72150 | 0.00897 |
| Mixed | 0.09473 | -0.33502 | 0.52449 | 0.21861 | 0.43336 | 0.66499 | 0.09473 | -0.17954 | 0.36901 | 0.13648 | 0.69411 | 0.49089 |
| Not specified | 0.63814 | 0.15778 | 1.11851 | 0.23904 | 2.66963 | 0.01027 | 0.63814 | -0.15001 | 1.42629 | 0.39220 | 1.62710 | 0.11013 |
| Other | -0.05224 | -0.69371 | 0.58924 | 0.31921 | -0.16364 | 0.87069 | -0.05224 | -0.77918 | 0.67471 | 0.36174 | -0.14440 | 0.88578 |
| Other ungulates | 0.12068 | -0.19647 | 0.43784 | 0.16133 | 0.74805 | 0.45487 | 0.12068 | -0.11837 | 0.35974 | 0.11896 | 1.01451 | 0.31532 |
| Plot_size | 0.00021 | -0.00031 | 0.00073 | 0.00027 | 0.78268 | 0.43427 | 0.00021 | -0.00026 | 0.00068 | 0.00024 | 0.88634 | 0.37976 |
| Year | 0.00119 | -0.01442 | 0.01680 | 0.00777 | 0.15313 | 0.87893 | 0.00119 | -0.01600 | 0.01838 | 0.00855 | 0.13912 | 0.88993 |
| root_inv_ESS | 0.65997 | 0.06287 | 1.25707 | 0.30403 | 2.17075 | 0.03034 | 0.65997 | -0.10294 | 1.42289 | 0.37964 | 1.73841 | 0.08842 |

## **Table S23.** Landscape scale MLMR – control method efficacy through time. Note that there were insufficient effect sizes in our complete case analysis to model how Neighbour effects retain their efficacy over time. There were sufficient effect sizes in our imputed case sensitivity analysis, hence why Neighbour effects and Neighbour effects:Months are blank for the Complete case and Robust analyses but not for the Imputed case.

|  | Complete case | | | | | | Robust | | | | Imputed case | | | |
| --- | --- | --- | --- | --- | --- | --- | --- | --- | --- | --- | --- | --- | --- | --- |
| term | est | ci.lb | ci.ub | se | stat | p.val | est | se | stat | p.val | est | se | stat | p.val |
| intrcpt | 0.39210 | 0.12970 | 0.65450 | 0.12653 | 3.09899 | 0.00524 | 0.39210 | 0.13367 | 2.93330 | 0.00553 | 0.50277 | 0.13975 | 3.59753 | 0.00035 |
| Natural debris | 0.02910 | -0.79398 | 0.85217 | 0.41855 | 0.06952 | 0.94462 | 0.02910 | 0.17978 | 0.16184 | 0.87224 | -0.10656 | 0.50147 | -0.21250 | 0.83197 |
| Neighbour effects |  |  |  |  |  |  |  |  |  |  | 0.45151 | 0.43359 | 1.04134 | 0.29858 |
| Repellents | 0.18391 | -0.37197 | 0.73979 | 0.28268 | 0.65060 | 0.51571 | 0.18391 | 0.30469 | 0.60360 | 0.54952 | 0.18928 | 0.28260 | 0.66978 | 0.50342 |
| Tree guards | 0.27351 | -0.22539 | 0.77240 | 0.25370 | 1.07808 | 0.28171 | 0.27351 | 0.27171 | 1.00662 | 0.32017 | 0.16833 | 0.28106 | 0.59890 | 0.54967 |
| Months | 0.00239 | 0.00064 | 0.00414 | 0.00089 | 2.68567 | 0.00746 | 0.00239 | 0.00068 | 3.50104 | 0.00115 | 0.00095 | 0.00115 | 0.82293 | 0.41091 |
| Plot_size | -0.00000 | -0.00055 | 0.00054 | 0.00028 | -0.01301 | 0.98963 | -0.00000 | 0.00033 | -0.01094 | 0.99133 | -0.00001 | 0.00006 | -0.12985 | 0.89704 |
| Natural debris:Months | -0.06276 | -0.09626 | -0.02927 | 0.01705 | -3.68081 | 0.00026 | -0.06276 | 0.00146 | -42.88846 | 0.00000 | -0.02016 | 0.06578 | -0.30651 | 0.76228 |
| Neighbour effects:Months |  |  |  |  |  |  |  |  |  |  | -0.01321 | 0.02544 | -0.51919 | 0.60529 |
| Repellents:Months | -0.00534 | -0.02522 | 0.01454 | 0.01012 | -0.52769 | 0.59793 | -0.00534 | 0.00515 | -1.03791 | 0.30555 | -0.00401 | 0.01630 | -0.24612 | 0.80567 |
| Tree guards:Months | 0.00040 | -0.00636 | 0.00717 | 0.00344 | 0.11668 | 0.90716 | 0.00040 | 0.00408 | 0.09856 | 0.92198 | 0.00232 | 0.00361 | 0.64197 | 0.52112 |

## **Table S24.** Landscape scale MLMR – control method efficacy through time publication bias.

|  | Complete case | | | | | | Robust | | | | | |
| --- | --- | --- | --- | --- | --- | --- | --- | --- | --- | --- | --- | --- |
| term | est | ci.lb | ci.ub | se | stat | p.val | est | ci.lb | ci.ub | se | stat | p.val |
| intrcpt | 0.35342 | 0.04907 | 0.65777 | 0.14591 | 2.42226 | 0.02505 | 0.35342 | 0.02360 | 0.68324 | 0.16292 | 2.16923 | 0.03639 |
| Natural debris | 0.04137 | -0.79072 | 0.87346 | 0.42312 | 0.09777 | 0.92217 | 0.04137 | -0.30459 | 0.38734 | 0.17090 | 0.24208 | 0.81002 |
| Repellents | 0.17726 | -0.41032 | 0.76485 | 0.29879 | 0.59327 | 0.55337 | 0.17726 | -0.42817 | 0.78270 | 0.29907 | 0.59272 | 0.55688 |
| Tree guards | 0.27459 | -0.24255 | 0.79173 | 0.26297 | 1.04418 | 0.29710 | 0.27459 | -0.26790 | 0.81708 | 0.26798 | 1.02468 | 0.31199 |
| Months | 0.00227 | 0.00048 | 0.00407 | 0.00092 | 2.48427 | 0.01328 | 0.00227 | 0.00060 | 0.00395 | 0.00083 | 2.74666 | 0.00915 |
| Plot_size | -0.00002 | -0.00056 | 0.00053 | 0.00028 | -0.05551 | 0.95576 | -0.00002 | -0.00068 | 0.00065 | 0.00033 | -0.04734 | 0.96249 |
| Year | -0.00245 | -0.02226 | 0.01736 | 0.00979 | -0.25042 | 0.80361 | -0.00245 | -0.02213 | 0.01723 | 0.00972 | -0.25201 | 0.80239 |
| root_inv_ESS | 0.15363 | -0.35725 | 0.66451 | 0.26008 | 0.59071 | 0.55496 | 0.15363 | -0.49221 | 0.79948 | 0.31903 | 0.48156 | 0.63288 |
| Natural debris:Months | -0.06293 | -0.09642 | -0.02944 | 0.01705 | -3.69094 | 0.00025 | -0.06293 | -0.06608 | -0.05978 | 0.00156 | -40.43701 | 0.00000 |
| Repellents:Months | -0.00516 | -0.02519 | 0.01487 | 0.01020 | -0.50589 | 0.61314 | -0.00516 | -0.01533 | 0.00501 | 0.00502 | -1.02681 | 0.31100 |
| Tree guards:Months | 0.00031 | -0.00646 | 0.00709 | 0.00345 | 0.09125 | 0.92733 | 0.00031 | -0.00799 | 0.00861 | 0.00410 | 0.07677 | 0.93921 |

## **Table S25.** Plant scale MLMA heterogeneity. CV = coefficient of variation; M = transformed coefficient of variation.

| Test | Total | Paper_ID | Cohort_ID | Plant_genus | OLRE |
| --- | --- | --- | --- | --- | --- |
| I^2^ | 90.17293 | 37.57919 | 34.42672 | 0.00000 | 18.16700 |
| CV | 1.03088 | 0.66549 | 0.63697 | 0.00018 | 0.46271 |
| M | 0.63838 | 0.24065 | 0.23034 | 0.00006 | 0.16732 |

## **Table S26.** Site scale MLMA heterogeneity.

| Test | Total | Paper_ID | Cohort_ID | Plant_genus | OLRE |
| --- | --- | --- | --- | --- | --- |
| I^2^ | 96.83244 | 14.89537 | 38.72042 | 17.79682 | 25.41983 |
| CV | 2.01333 | 0.78964 | 1.27313 | 0.86313 | 1.03155 |
| M | 0.79828 | 0.15928 | 0.25681 | 0.17411 | 0.20808 |

## **Table S27.** Landscape scale MLMA heterogeneity.

| Test | Total | Paper_ID | Cohort_ID | Plant_genus | OLRE |
| --- | --- | --- | --- | --- | --- |
| I^2^ | 96.79560 | 18.74397 | 72.64076 | 0.00000 | 5.41087 |
| CV | 1.79053 | 0.78792 | 1.55111 | 0.00008 | 0.42334 |
| M | 0.73422 | 0.20942 | 0.41226 | 0.00002 | 0.11252 |

**Appendix S2. Meta-analyses reference list**

All references here had data extracted that were used in our meta-analyses.

Abraham, E., Sklavou, P., Loufi, A., Parissi, Z. & Kyriazopoulos, A. (2018). The effect of combined herbivory by wild boar and small ruminants on the regeneration of a deciduous oak forest. *Forests* **9**, 580.

Adams, S.N. (1975). Sheep and cattle grazing in forests: a review. *Journal of Applied Ecology* **12**, 143–152.

Allan, G.G., Gustafson, D.I., Mikels, R.A., Miller, J.M. & Neogi, S. (1984). Reduction of deer browsing of Douglas-fir (*Pseudotsuga menziesii*) seedlings by quadrivalent selenium. *Forest Ecology and Management* **7**, 163–181.

Andelt, W.F., Burnham, K.P. & Baker, D.L. (1994). Effectiveness of capsaicin and bitrex repellents for deterring browsing by captive mule deer. *The Journal of Wildlife Management* **58**, 330.

Andelt, W.F., Burnham, K.P. & Manning, J.A. (1991). Relative effectiveness of repellents for reducing mule deer damage. *The Journal of Wildlife Management*, **55** 341.

Anderson, C.E., Chapman, K.A., White, M.A. & Cornett, M.W. (2002). Effects of browsing control on establishment and recruitment of eastern white pine (*Pinus strobus L.*) at Cathedral Grove, Lake Superior Highlands, Minnesota, USA. *Natural Areas Journal* **22**, 202–210.

Andruk, C.M., Schwope, C. & Fowler, N.L. (2014). The joint effects of fire and herbivory on hardwood regeneration in central Texas woodlands. *Forest Ecology and Management* **334**, 193–200.

Asnani, K.M., Klips, R.A. & Curtis, P.S. (2006). Regeneration of woodland vegetation after deer browsing in Sharon Woods Metro Park, Franklin County, Ohio. *Ohio Journal of Science* **106**, 86–92.

Baker, B.W., Peinetti, H.R. & Coughenour, M.B. (2005). Resilience of willow stems after release from intense elk browsing. *Rangeland Ecology & Management*, **58** 575–581.

Barrere, J., Petersson, L.K., Boulanger, V., Collet, C., Felton, A.M., Löf, M. & Saïd, S. (2021). Canopy openness and exclusion of wild ungulates act synergistically to improve oak natural regeneration. *Forest Ecology and Management* **487**, 118976.

Barton, C., Miller, J., Sena, K., Angel, P. & French, M. (2015). Evaluating the use of tree shelters for direct seeding of castanea on a surface mine in Appalachia. *Forests* **6**, 3514–3527.

Basey, J.M., Jenkins, S.H. & Miller, G.C. (1990). Food selection by beavers in relation to inducible defenses of *Populus tremuloides*. *Oikos* **59**, 57–62.

Beguin, J., Pothier, D. & Prévost, M. (2009). Can the impact of deer browsing on tree regeneration be mitigated by shelterwood cutting and strip clearcutting? *Forest Ecology and Management* **257**, 38–45.

Bergquist, J., Löf, M. & Örlander, G. (2009). Effects of roe deer browsing and site preparation on performance of planted broadleaved and conifer seedlings when using temporary fences. *Scandinavian Journal of Forest Research* **24**, 308–317.

Bergquist, J. & Örlander, G. (1996). Browsing deterrent and phytotoxic effects of roe deer repellents on *Pinus sylvestris* and *Picea abies* seedlings. *Scandinavian Journal of Forest Research* **11**, 145–152.

Bergvall, U.A. & Leimar, O. (2017). Directional associational plant defense from Red deer (*Cervus elaphus*) foraging decisions. *Ecosphere*, **8**, e01714.

Beringer, J., Hansen, L.P., Heinen, R.A. & Giessman, N.F. (1994). Use of Dogs to Reduce Damage by Deer to a White Pine Plantation. *Wildlife Society Bulletin (1973-2006)*, **22**, 627–632.

Black, H. & Vladimiroff, B. (1963). Effect of grazing on regeneration of Douglas-fir in southwestern Oregon. *Proceedings Society of American Forestry* **1963**, 69–76.

Blackhall, M., Raffaele, E. & Veblen, T.T. (2008). Cattle affect early post-fire regeneration in a *Nothofagus dombeyi*–*Austrocedrus chilensis* mixed forest in northern Patagonia, Argentina. *Biological Conservation*, **141**, 2251–2261.

Bolibok, L., Andrzejczyk, T., Szeligowski, H. & Liziniewicz, M. (2021). New methods of oak planting require modification of tending prescriptions under high browsing pressure – A case study from north-eastern Poland. *Forest Ecology and Management*, **497**, 119449.

Boyer, W.D. (1967). Grazing hampers development of longleaf pine seedlings in southwest Alabama. *Journal of Forestry*, **65**, 336–338.

Breton, V., Forestier, O., Guindon, O. & Evette, A. (2014). Ecological restoration under pressure from invasive animal species: use of Salicaceae cuttings in a river bank overrun by coypu. *River Research and Applications* **30**, 1002–1012.

Brousseau, M., Thiffault, N., Beguin, J., Roy, V. & Tremblay, J.-P. (2017). Deer browsing outweighs the effects of site preparation and mechanical release on balsam fir seedlings performance: Implications to forest management. *Forest Ecology and Management* **405**, 360–366.

Bulinski, J. & McArthur, C. (1999). An experimental field study of the effects of mammalian herbivore damage on *Eucalyptus nitens* seedlings. *Forest Ecology and Management* **113**, 241–249.

Candaele, R., Ligot, G., Licoppe, A., Lievens, J., Fichefet, V., Jonard, M., André, F. & Lejeune, P. (2023). Interspecific growth reductions caused by wild ungulates on tree seedlings and their implications for temperate quercus-fagus forests. *Forests* **14**, 1330.

Casabon, C. & Pothier, D. (2008). Impact of deer browsing on plant communities in cutover sites on Anticosti Island. *Écoscience* **15**, 389–397.

Case, R.L. & Kauffman, J.B. (1997). Wild ungulate influences on the recovery of willows, black cottonwood and thin-leaf alder following cessation of cattle grazing in northeastern Oregon. *Northwest science* **71**, 115–126.

Clements, D.R., Luginbill, S., Jordan, D.A., Dragt, R.V. & Pelant, R.K. (2011). Techniques to promote garry oak seedling growth and survival in areas with high levels of herbivory and competition. *Northwest Science* **85**, 172–181.

Coggins, S.T. & Conover, M.R. (2005). Effect of pocket gophers on aspen regeneration. *Journal of Wildlife Management* **69**, 752–759.

Conover, M.R. & Kania, G.S. (1987). Effectiveness of human hair, BGR, and a mixture of blood meal and peppercorns in reducing deer damage to young apple trees. In: *Third Eastern Wildlife Damage Control Conference*, p. 10.

Cooke, A.S. & Lakhani, K.H. (1996). Damage to coppice regrowth by muntjac deer *Muntiacus reevesi* and protection with electric fencing. *Biological Conservation* **75**, 231–238.

Curtis, P.D. & Boulanger, J.R. (2010). Relative effectiveness of repellents for preventing deer damage to Japanese yews. *HortTechnology* **20**, 730–734.

Curtis, P.D. & Eshenaur, B.C. (2022). Trico: A novel repellent for preventing deer damage to ornamental shrubs. *Human–Wildlife Interactions* **16**, 22–28.

den Herder, M., Virtanen, R. & Roininen, H. (2008). Reindeer herbivory reduces willow growth and grouse forage in a forest-tundra ecotone. *Basic and Applied Ecology* **9**, 324–331.

Dietz, D.R. & Tigner, J.R. (1968). Evaluation of two mammal repellents applied to browse species in the Black Hills. *The Journal of Wildlife Management* **32**, 109–114.

Drexhage, M. & Colin, F. (2003). Effects of browsing on shoots and roots of naturally regenerated sessile oak seedlings. *Annals of Forest Science* **60**, 173–178.

El Kateb, H., Benabdellah, B., Ammer, C. & Mosandl, R. (2004). Reforestation with native tree species using site preparation techniques for the restoration of woodlands degraded by air pollution in the Erzgebirge, Germany. *European Journal of Forest Research* **123**, 117–126.

Elie, J.-G., Ruel, J.-C. & Lussier, J.-M. (2009). Effect of browsing, seedbed, and competition on the development of yellow birch seedlings in high-graded stands. *Northern Journal of Applied Forestry* **26**, 99–105.

Forrester, J.A., Leopold, D.J. & Underwood, H.B. (2006). Isolating the effects of white-tailed deer on the vegetation dynamics of a rare maritime American holly forest. *The American Midland Naturalist*, **156**, 135–150.

Forrester, J.A., Lorimer, C.G., Dyer, J.H., Gower, S.T. & Mladenoff, D.J. (2014). Response of tree regeneration to experimental gap creation and deer herbivory in north temperate forests. *Forest Ecology and Management*, **329**, 137–147.

García, D. & Ramón Obeso, J. (2003). Facilitation by herbivore-mediated nurse plants in a threatened tree, *Taxus baccata*: local effects and landscape level consistency. *Ecography* **26**, 739–750.

George, J.F. & Powell, J. (1977). Deer browsing and browse production of fertilized American elm sprouts. *Journal of Range Management* **30**, 357.

Gillespie, A.R., Rathfon, R. & Myers, R.K. (1996). Rehabilitating a young northern red oak planting with tree shelters. *Northern Journal of Applied Forestry* **13**, 24–29.

Gillingham, M.P., Speyer, M.R., Northway, S. & McLaughlin, R. (1987). Feeding preference and its relation to herbivore repellent studies. *Canadian Journal of Forest Research* **17**, 146–149.

Gong, Y.L., Swaine, M.D. & Miller, H.G. (1991). Effects of fencing and ground preparation on natural regeneration of native pinewood over 12 years in Glen Tanar, Aberdeenshire. *Forestry: An International Journal of Forest Research* **64**, 157–168.

Gourley, M., Vomocil, M. & Newton, M. (1990). Forest weeding reduces the effect of deer-browsing on Douglas fir. *Forest Ecology and Management* **36**, 177–185.

Grant, L.S., McArthur, C., Banks, P.B., Possell, M. & Price, C.J. (2024). Visit, investigate, ignore: Olfactory misinformation reduces browsing damage to valued seedlings by a mammalian herbivore. *Biological Conservation* **291**, 110465.

Graveline, D.W. (1997). Growth and protection of selected northern red oak planted on old field sites in Tennessee. Master's Thesis, University of Tennessee, <https://trace.tennessee.edu/utk_gradthes/6770>

Grisez, T.J. (1960). Slash helps protect seedlings from deer browsing. *Journal of Forestry* ***58***(5), 385–387*.*

Haines, P., Bell, A. & Thatcher, L. (1994). Evaluation of some factors involved in reducing browsing damage to eucalypt trees by sheep. *Australian Journal of Experimental Agriculture* **34**, 601.

Hardenbol, A.A., den Herder, M. & Kouki, J. (2021). Long-term effects of prescribed burning, tree retention, and browsing on deciduous tree recruitment in European boreal forests. *Canadian Journal of Forest Research* **51**, 660–667.

Hasstedt, S.L. & Annighöfer, P. (2020). Initial survival and development of planted European beech (*Fagus sylvatica L*.) and small-leaved lime (*Tilia cordata Mill*.) seedlings competing with black cherry (*Prunus serotina Ehrh*.). *Plants* **9**, 677.

Hjelm, K., Mc Carthy, R. & Rytter, L. (2018). Establishment strategies for poplars, including mulch and plant types, on agricultural land in Sweden. *New Forests* **49**, 737–755.

Horner, G.J., Cunningham, S.C., Thomson, J.R., Baker, P.J. & Mac Nally, R. (2016). Recruitment of a keystone tree species must concurrently manage flooding and browsing. *Journal of Applied Ecology* **53**, 944–952.

Horsley, S.B. & Marquis, D.A. (1983). Interference by weeds and deer with Allegheny hardwood reproduction. *Canadian Journal of Forest Research* **13**, 61–69.

Huertas Herrera, A., Promis, Á., Toro-Manríquez, M., Lencinas, M.V., Martínez Pastur, G. & Río, M. (2022). Rehabilitation of *Nothofagus pumilio* forests in Chilean Patagonia: can fencing and planting season effectively protect against exotic European hare browsing? *New Forests* **53**, 469–485.

Huffman, A. (2022). Effects of light availability and herbivory on planted white oak (*Quercus alba*) seedlings and acorns. PhD thesis, University of Missouri-Columbia.

Hunn, J. (2007). Retention of logging debris to reduce deer browsing and promote forest regeneration. Honors thesis, Cornell University

Husheer, S.W., Hansen, Q.W. & Urlich, S.C. (2005). Effects of red deer on tree regeneration and growth in Aorangi Forest, Wairarapa. *New Zealand Journal of Ecology* **29**, 271–277.

Jacques, R., Stovall, J., Comer, C., Williams, H. & Symmank, M. (2021). Herbivory and flooding impacts on planted bottomland hardwood seedlings. *Forestry: An International Journal of Forest Research* **95,** 215–228.

Jensen, A.M., Götmark, F. & Löf, M. (2012). Shrubs protect oak seedlings against ungulate browsing in temperate broadleaved forests of conservation interest: A field experiment. *Forest Ecology and Management* **266**, 187–193.

Keeton, W.S. (2008). Evaluation of tree seedling mortality and protective strategies in riparian forest restoration. *Northern Journal of Applied Forestry* **25**, 117–123.

Kimball, B.A. & Nolte, D.L. (2006). Development of a new deer repellent for the protection of forest resources. *Western Journal of Applied Forestry* **21**, 108–111.

Kimball, B.A., Nolte, D.L. & Perry, K.B. (2005). Hydrolyzed casein reduces browsing of trees and shrubs by white-tailed deer. *HortScience* **40**, 1810–1814.

Kimball, B.A. & Perry, K.R. (2009). Evaluating new protein sources for development of a deer repellent product. *Crop Protection* **28**, 364–366.

Kimball, B.A., Russell, J.H., DeGraan, J.P. & Perry, K.R. (2008). Screening hydrolyzed casein as a deer repellent for reforestation applications. *Western Journal of Applied Forestry* **23**, 172–176.

Kisanuki, H., Nakai, A., Nadamoto, A. & Wakino, M. (2009). Pits and rocky microsites in a subalpine forest stand facilitate regeneration of spruce saplings by suppressing dwarf bamboo growth inside a deer-proof fence. *Journal of Forest Research* **14**, 342–348.

Knowles, R.L. & Tahau, F. (1979). Repellent to protect radiata pine seedlings from browsing by sheep. *New Zealand Journal of Forestry Science* **9**, 3–9.

Kochenderfer, J.N. & Ford, W.M. (2008). Utility of wire cages, tree shelters, and repellents to minimize herbivory to oak by white-tailed deer. *Research paper NRS-5. US Department of Agriculture, Forest Service, Northern Research Station*. <https://research.fs.usda.gov/download/treesearch/14585.pdf>

Konôpka, B., Šebeň, V., Pajtík, J. & Shipley, L.A. (2021). Excluding large wild herbivores reduced Norway spruce dominance and supported tree species richness in a young, naturally regenerated stand. *Forests* **12**, 737.

Kota, A.M. & Bartos, D.L. (2010). Evaluation of techniques to protect aspen suckers from ungulate browsing in the Black Hills. *Western Journal of Applied Forestry* **25**, 161–168.

Kuiters, A.T. & Slim, P.A. (2002). Regeneration of mixed deciduous forest in a Dutch forest-heathland, following a reduction of ungulate densities. *Biological Conservation* **105**, 65–74.

Kýpeťová, M., Walas, Ł., Jaloviar, P. & Iszkuło, G. (2017). Influence of herbivory pressure on the growth rate and needle morphology of *Taxus baccata L*. juveniles. *Dendrobiology* **79**, 10–19.

Laurent, L., Mårell, A., Balandier, P., Holveck, H. & Saïd, S. (2017). Understory vegetation dynamics and tree regeneration as affected by deer herbivory in temperate hardwood forests. *iForest - Biogeosciences and Forestry* **10**, 837–844.

Lehtonen, J. & Heikkinen, R.K. (1995). On the recovery of mountain birch after Epirrita damage in Finnish Lapland, with a particular emphasis on reindeer grazing. *Écoscience* **2**, 349–356.

Leonardsson, J., Löf, M. & Götmark, F. (2015). Exclosures can favour natural regeneration of oak after conservation-oriented thinning in mixed forests in Sweden: A 10-year study. *Forest Ecology and Management* **354**, 1–9.

Lindmark, M., Sunnerheim, K. & Jonsson, B.G. (2020). Natural browsing repellent to protect Scots pine *Pinus sylvestris* from European moose *Alces alces*. *Forest Ecology and Management* **474**, 118347.

Löf, M., Barrere, J., Engman, M., Petersson, L.K. & Villalobos, A. (2021). The influence of fencing on seedling establishment during reforestation of oak stands: a comparison of artificial and natural regeneration techniques including costs. *European Journal of Forest Research* **140**, 807–817.

Love, B.E., Bork, E.W. & Spaner, D. (2009). Tree seedling establishment in living fences: a low-cost agroforestry management practice for the tropics. *Agroforestry Systems* **77**, 1–8.

MacGowan, B. J., Severeid, L., & Skemp, J. (2004). Control of deer damage with chemical repellents in regenerating hardwood stands. (eds. Michler, C.H., Pijut, P.M., Van Sambeek, J.W., Coggeshall, M.V., Seifert, J., Woeste, K., Overton, R., Ponder, F.). In: *Proceedings of the 6th Walnut Council Research Symposium; Gen. Tech. Rep. NC-243. St. Paul, MN: US Department of Agriculture, Forest Service, North Central Research Station.* **243**, 127–133.

Martínez Pastur, G., Soler, R., Ivancich, H., Lencinas, M.V., Bahamonde, H. & Peri, P.L. (2016). Effectiveness of fencing and hunting to control *Lama guanicoe* browsing damage: Implications for *Nothofagus pumilio* regeneration in harvested forests. *Journal of Environmental Management* **168**, 165–174.

Maschinski, J. (2001). Impacts of ungulate herbivores on a rare willow at the southern edge of its range. *Biological Conservation* **101**, 119–130.

Massei, G. & Hartley, S.E. (2000). Disarmed by domestication? Induced responses to browsing in wild and cultivated olive. *Oecologia* **122**, 225–231.

Matney, C.A., Boyd, C.S. & Stringham, T.K. (2005). Use of felled junipers to protect streamside willows from browsing. *Rangeland Ecology & Management* **58**, 652–655.

McClenahen, J. & Hutnik, R. (1979). Effects of cutting intensity, deer browsing, and liming on Allegheny hardwood tree regeneration. *Canadian Journal of Forest Research* **9**, 362–368.

McLaren, B., Hermanutz, L., Gosse, J., Collet, B. & Kasimos, C. (2009). Broadleaf competition interferes with balsam fir regeneration following experimental removal of moose. *Forest Ecology and Management* **257**, 1395–1404.

Miller, A.M., McArthur, C. & Smethurst, P.J. (2006). Characteristics of tree seedlings and neighbouring vegetation have an additive influence on browsing by generalist herbivores. *Forest Ecology & Management* **228**, 197–205.

Miller, A.M., O'Reilly-Wapstra, J.M., Fitzgerald, H.P., Paterson, S.C., Stam, L., Walsh, A., Wardlaw, T. & Potts, B.M. (2008). Effectiveness of repellents for reducing damage to eucalypt seedlings by browsing mammals. *Australian Forestry* **71**, 303–310.

Miller, A.M., O’Reilly-Wapstra, J.M., Potts, B.M. & McArthur, C. (2011). Repellent and stocking guards reduce mammal browsing in eucalypt plantations. *New Forests: International Journal on the Biology, Biotechnology, and Management of Afforestation and Reforestation* **42**, 301–316.

Miller, G.W., Brose, P.H. & Gottschalk, K.W. (2016). Advanced oak seedling development as influenced by shelterwood treatments, competition control, deer fencing, and prescribed fire. *Journal of Forestry* **115**, 179–189.

Milunas, M.C., Rhoads, A.F. & Russell Mason, J. (1994). Effectiveness of odour repellents for protecting ornamental shrubs from browsing by white-tailed deer. *Crop Protection* **13**, 393–397.

Moran, S. (1996). Efficacy of the repellent RO‐PEL® in reducing damage by the rock hyrax (*Procavia capensis*) to fruit trees. *International Journal of Pest Management* **42**, 273–276.

Morimoto, J., Sugiura, M., Morimoto, M. & Nakamura, F. (2021). Restoration of natural forests after severe wind disturbance in a cold, snowy region with a deer population: implications from 15 years of field experiments. *Frontiers in Forests and Global Change* **4**, 675475.

Moser, B.W. (2000). A comparison of physical, chemical, and genetic controls to reduce deer browse damage to hybrid poplar seedlings. In: *Proceedings of the Vertebrate Pest Conference* **19**, 88–92.

Moser, B.W. (2002). An evaluation of chemical repellents and Vexar bud caps to reduce winter deer and elk browsing on conifer seedlings in northern Idaho. In: *Proceedings of the Vertebrate Pest Conference* **20**, 326–329.

Moser, S. & Greet, J. (2018). Unpalatable neighbours reduce browsing on woody seedlings. *Forest Ecology and Management* **414**, 41–46.

Mower, K.J., Townsend, T.W. & Tyznik, W.J. (1997). White-tailed deer damage to experimental apple orchards in Ohio. *Wildlife Society Bulletin (1973-2006)* **25**, 337–343.

Mrnka, L., Frantík, T., Baldassarre Švecová, E., Schmidt, C.S., Vosátka, M., Knot, D., Šolcová, O. & Hart, V. (2021). Clone-dependent browsing damage of poplar plantations and the repellent potential of *Populus nigra* × *P. maximowiczii* ʻMax-4’. *Forest Ecology and Management*, **483**, 118888.

Murata, I., Saruki, S., Kubota, K., Inoue, S., Tashiro, N., Enoki, T., Utsumi, Y. & Inoue, S. (2009). Effects of sika deer (*Cervus nippon*) and dwarf bamboo (*Sasamorpha borealis*) on seedling emergence and survival in cool-temperate mixed forests in the Kyushu Mountains. *Journal of Forest Research*, **14**, 296–301.

Nilar, H., Maute, K., Dawson, M.J., Scarborough, R., Hudson, J., Reay, J. & Gooden, B. (2019). Effectiveness of different herbivore exclusion strategies for restoration of an endangered rainforest community. *Forest Ecology and Management*, **435**, 18–26.

Nolte, D.L. (1998). Efficacy of selected repellents to deter deer browsing on conifer seedlings. *International biodeterioration & biodegradation* **42**, 101–107.

Nolte, D.L., Farley, J.P., Campbell, D.L., Epple, G.M. & Mason, J.R. (1993). Potential repellents to prevent mountain beaver damage. *Crop Protection* **12**, 624–626.

Nordkvist, M., Klapwijk, M.J., Barets, S. & Björkman, C. (2023). The relationship between moose browsing, habitat structure and predation pressure on insect herbivores. *Basic and Applied Ecology* **66**, 1–10.

Owings, C.F., Jacobs, D.F., Shields, J.M., Saunders, M.R. & Jenkins, M.A. (2017). Individual and interactive effects of white-tailed deer and an exotic shrub on artificial and natural regeneration in mixed hardwood forests. *AoB PLANTS* **9**, plx024.

Palmer, W.L., Wingard, R.G. & George, J.L. (1983). Evaluation of white-tailed deer repellents. *Wildlife Society Bulletin (1973-2006)* **11**, 164–166.

Parsons, M.H., Lamont, B.B., Koch, J.M. & Dods, K. (2007). Disentangling competition, herbivory, and seasonal effects on young plants in newly restored communities. *Restoration Ecology* **15**, 250–262.

Pellerin, M., Saïd, S., Richard, E., Hamann, J.-L., Dubois-Coli, C. & Hum, P. (2010). Impact of deer on temperate forest vegetation and woody debris as protection of forest regeneration against browsing. *Forest Ecology and Management* **260**, 429–437.

Perles, S.J., Niu, X.M., Ruth, A.D. & Gibbons, L.D. (2021). Initial conditions influence effects of prescribed burns and deer exclosure fences on tree regeneration and understory diversity in Appalachian oak-dominated forests. *Forest Ecology and Management* **495**, 119353.

Petersson, L.K., Dey, D.C., Felton, A.M., Gardiner, E.S. & Löf, M. (2020). Influence of canopy openness, ungulate exclosure, and low‐intensity fire for improved oak regeneration in temperate Europe. *Ecology and Evolution* **10**, 2626–2637.

Pfeffer, S.E., Singh, N.J., Cromsigt, J.P.G.M. & Widemo, F. (2021). Summer and winter browsing affect conifer growth differently: An experimental study in a multi-species ungulate community. *Forest Ecology and Management* **494**, 119314.

Pinna, S., Malenfant, A. & Côté, M. (2012). Vigor and growth responses of sugar maple and yellow birch seedlings according to different competing vegetation types and fabric shelter use. *Northern Journal of Applied Forestry* **29**, 133–140.

Porter, W.F. (1983). A baited electric fence for controlling deer damage to orchard seedlings. *Wildlife Society Bulletin (1973-2006)* **11**, 325–327.

Prach, K., Lepš, J. & Michálek, J. (1996). Establishment of *Picea abies* seedlings in a central European mountain grassland: an experimental study. *Journal of Vegetation Science* **7**, 681–684.

Puettmann, K. J., Cole, L., & Newton, M. (2024). Deer browse deterrents and microsite selection influence *Callitropsis nootkatensis* and *Thuja plicata* planting success in Southeast Alaska. *New Forests* **55**, 877–895.

Rangen, S.A., Hawley, A.W.L. & Hudson, R.J. (1993). Acceptability of thiram-treated conifers to snowshoe hares. *Canadian Journal of Forest Research* **23**, 1314–1320.

Rangen, S.A., Hawley, A.W.L. & Hudson, R.J. (1993). Palatability of Anispray®-treated white spruce to snowshoe hares. *Canadian Journal of Forest Research* **23**, 1321–1324.

Rangen, S.A., Hawley, A.W.L. & Hudson, R.J. (1993). Response of Captive Snowshoe Hares to Thiram-Treated Conifers. *The Journal of Wildlife Management* **57**, 648.

Read, J., Guerin, J., Duval, D. & Moseby, K. (2021). Charred and chewed chalkies: Effects of fire and herbivory on the reintroduction of an endangered wattle. *Ecological Management & Restoration* **22**, 35–43.

Rediske, J.H. & Lawrence, W.H. (1962). Selenium as a wildlife repellent for Douglas-fir seedlings. *Forest Science* **8**, 142–148.

Reikowski, E.S., Refsland, T. & Cushman, J.H. (2022). Ungulate herbivores as drivers of aspen recruitment and understory composition throughout arid montane landscapes. *Ecosphere* **13**, e4225.

Romagosa, M.A. & Robison, D.J. (2003). Biological constraints on the growth of hardwood regeneration in upland Piedmont forests. *Forest Ecology and Management* **175**, 545–561.

Royo, A.A., Pinchot, C.C., Stanovick, J.S. & Stout, S.L. (2019). Timing is not everything: assessing the efficacy of pre- versus post-harvest herbicide applications in mitigating the burgeoning birch phenomenon in regenerating hardwood stands. *Forests* **10**, 324.

Russell, F.L. & Fowler, N.L. (2004). Effects of white-tailed deer on the population dynamics of acorns, seedlings and small saplings of *Quercus buckleyi*. *Plant Ecology (formerly Vegetatio)* **173**, 59–72.

Santilli, F., Mori, L. & Galardi, L. (2004). Evaluation of three repellents for the prevention of damage to olive seedlings by deer. *European Journal of Wildlife Research* **50**, 85–89.

Scowcroft, P.G. & Hobdy, R. (1987). Recovery of goat-damaged vegetation in an insular tropical montane forest. *Biotropica* **19**, 208–215.

Shimoda, K., Kimura, K., Kanzaki, M. & Yoda, K. (1994). The regeneration of pioneer tree species under browsing pressure of Sika deer in an evergreen oak forest. *Ecological Research* **9**, 85–92.

Smallidge, P.J., Chedzoy, B., Curtis, P. & Sims, K. (2021). Evaluating the construction and effectiveness of slash walls at the perimeter of regeneration harvests to exclude deer. *Forest Ecology and Management* **497**, 119529.

Smola, M., Cafourek, J., Kusbach, A., Šenfeldr, M., Střítecký, J. & Maděra, P. (2023). Impact of protection methods and abiotic factors on *Nothofagus pumilio* seedlings mortality in Torres del Paine National Park, Chile. *Journal of Forest Science* **69**, 389–400.

Stange, E.E. & Shea, K.L. (1998). Effects of deer browsing, fabric mats and tree shelters on *Quercus rubra* seedlings. *Restoration Ecology* **6**, 29–34.

Stapleton, J.P., Ikin, K. & Freudenberger, D. (2017). Coarse woody debris can reduce mammalian browsing damage of woody plant saplings in box-gum grassy woodlands. *Ecological Management & Restoration* **18**, 223.

Strohmeyer, S. & Maschinski, J. (1996). Cattle and elk herbivory on Arizona willow (*Salix arizonica*). (technical coordinators: Maschinski, J., Hammond, H.D. & Holter, L.), In: *Southwestern rare and endangered plants. Proceedings of the second conference. USDA Forest Service General Technical Report RM-GTR-283*, pp. 187–192.

Stutz, R.S., Banks, P.B., Dexter, N. & McArthur, C. (2015). Herbivore search behaviour drives associational plant refuge. *Acta Oecologica* **67**, 1–7.

Stutz, R.S., Croak, B.M., Leimar, O. & Bergvall, U.A. (2017). Borrowed plant defences: Deterring browsers using a forestry by-product. *Forest Ecology and Management* **390**, 1–7.

Stutz, R.S., Pedersen, S., Teräväinen, M., Kjellander, P., Leimar, O., Verschuur, L. & Bergvall, U.A. (2019). Efficient application of a browsing repellent: Can associational effects within and between plants be exploited? *European Journal of Forest Research* **138**, 253–262.

Sullivan, T.P. & Crump, D.R. (1984). Influence of mustelid scent-gland compounds on suppression of feeding by snowshoe hares (*Lepus americanus*). *Journal of chemical ecology* **10**, 1809–1821.

Sullivan, T.P., Nordstrom, L.O. & Sullivan, D.S. (1985). Use of predator odors as repellents to reduce feeding damage by herbivores. *Journal of chemical ecology* **11**, 903–919.

Sweeney, B.W., Czapka, S.J. & Petrow, L.C.A. (2007). How planting method, weed abatement, and herbivory affect afforestation success. *Southern Journal of Applied Forestry* **31**, 85–92.

Sweeney, B.W., Czapka, S.J. & Yerkes, T. (2002). Riparian forest restoration: increasing success by reducing plant competition and herbivory. *Restoration Ecology* **10**, 392–400.

Swihart, R.K. & Conover, M.R. (1990). Reducing deer damage to yews and apple trees: testing Big Game Repellent®, RO pel®, and soap as repellents. *Wildlife Society Bulletin (1973-2006)* **18**, 156–162.

Swihart, R.K., Pignatello, J.J. & Mattina, M.J.I. (1991). Aversive responses of white-tailed deer, *Odocoileus virginianus*, to predator urines. *Journal of chemical ecology* **17**, 767–777.

Takatsuki, S. & Gorai, T. (1994). Effects of Sika deer on the regeneration of a *Fagus crenata* forest on Kinkazan Island, northern Japan. *Ecological Research* **9**, 115–120.

Taylor, T.S., Loewenstein, E.F. & Chappelka, A.H. (2006). Effect of animal browse protection and fertilizer application on the establishment of planted Nuttall oak seedlings. *New Forests* **32**, 133–143.

Thyroff, E.C., Burney, O.T., Oliet, J.A., Redick, C.H. & Jacobs, D.F. (2022). Toward identifying alternatives to fencing for forest restoration: tube shelters outperform mesh shelters for deer browse protection of live oak, *Quercus virginiana*. *Land* **11**, 966.

Tierson, W.C. (1969). Controlling deer use of forest vegetation with electric fences. *The Journal of Wildlife Management* **33**, 922.

Timmins, S.M. (2002). Impact of cattle on conservation land licensed for grazing in South Westland, New Zealand. *New Zealand Journal of Ecology* **26**, 107–120.

Truax, B., Fortier, J., Gagnon, D. & Lambert, F. (2021). Long-term effects of white-tailed deer overabundance, hybrid poplar genotype and planting stock type on tree growth and ecosystem services provision in bioenergy buffers. *Forest Ecology and Management* **480**, 118673.

Tuft, K.D., Crowther, M.S. & McArthur, C. (2012). Fire and grazing influence food resources of an endangered rock-wallaby. *Wildlife Research* **39**, 436.

Vacek, Z. (2017). Structure and dynamics of spruce-beech-fir forests in nature reserves of the Orlické hory Mts. in relation to ungulate game. *Central European Forestry Journal* **63**, 23–34.

Vacek, Z., Vacek, S., Bílek, L., Král, J., Remeš, J., Bulušek, D. & Králíček, I. (2014). Ungulate impact on natural regeneration in spruce-beech-fir Stands in Černý důl nature reserve in the Orlické Hory mountains, case study from central Sudetes. *Forests* **5**, 2929–2946.

Van Hees, A.F.M., Kuiters, A.T. & Slim, P.A. (1996). Growth and development of silver birch, pedunculate oak and beech as affected by deer browsing. *Forest Ecology and Management* **88**, 55–63.

Von Carlowitz, P. & Wolf, G. (1991). Potential and limitations of natural repellents against early destructive browsing by livestock and game. *Agroforestry Systems* **16**, 33–40.

Wagner, K.K. & Nolte, D.L. (2000). Evaluation of Hot Sauce® as a repellent for forest mammals. *Wildlife Society Bulletin (1973-2006)* **28**, 76–83.

Wagner, K.K. & Nolte, D.L. (2001). Comparison of active ingredients and delivery systems in deer repellents. *Wildlife Society Bulletin* **29**, 322–330.

Waller, D.M. & Maas, L.I. (2013). Do white-tailed deer and the exotic plant garlic mustard interact to affect the growth and persistence of native forest plants? *Forest Ecology and Management* **304**, 296–302.

Ward, J.S., Gent, M.P.N. & Stephens, G.R. (2000). Effects of planting stock quality and browse protection-type on height growth of northern red oak and eastern white pine. *Forest Ecology and Management* **127**, 205–216.

Yagi, T. (2022). The combined effects of tree shelters, large stock and vegetation control on the early growth of conifer seedlings. *Journal of Forest Research* **27**, 206–213.
